# Modelling welfare estimates in discrete choice experiments for seaweed-based renewable energy

**DOI:** 10.1371/journal.pone.0260352

**Published:** 2021-11-29

**Authors:** Petr Mariel, Simona Demel, Alberto Longo

**Affiliations:** 1 Department of Quantitative Methods, University of the Basque Country, Bilbao, Spain; 2 School of Biological Sciences, IGFS, Gibson Institute, Queen’s University Belfast, Belfast, United Kingdom; Groupe ESC Dijon Bourgogne, FRANCE

## Abstract

We explore what researchers can gain or lose by using three widely used models for the analysis of discrete choice experiment data—the random parameter logit (RPL) with correlated parameters, the RPL with uncorrelated parameters and the hybrid choice model. Specifically, we analyze three data sets focused on measuring preferences to support a renewable energy programme to grow seaweed for biogas production. In spite of the fact that all three models can converge to very similar median WTP values, they cannot be used indistinguishably. Each model is based on different assumptions, which should be tested before their use. The fact that standard sample sizes usually applied in environmental valuation are generally unable to capture the outcome differences between the models cannot be used as a justification for their indistinct application.

## Introduction

The analysis of discrete choice experiment (DCE) data for valuing environmental goods and resources has evolved dramatically in the last 25 years after their initial application to environmental economics [1]. The earliest model was the multinomial logit (MNL) model [2–4], which implies strict assumptions, such as the independence of irrelevant alternatives, uncorrelated attributes, and observable taste heterogeneity that can be explored, for example, with interaction terms between DCE attributes and respondents’ characteristics [5, 6]. To relax the assumptions of the MNL, the most widely used model (apart from the Latent Class Model) has been the mixed logit (MXL) model, mainly in the form of the random parameter logit (RPL) model [7]. The RPL model expands the MNL and requires researchers to decide (a) which parameters should be modeled as randomly distributed, (b) which mixing distributions for the random coefficients to adopt, and (c) whether the coefficients of the RPL should be correlated. Whilst (a) and (b) have received some attention by practitioners, researchers have most frequently assumed the coefficients of the RPL to be uncorrelated. However, this is a very limiting assumption that can be incorporated into the model only after an appropriate test. An RPL model with uncorrelated utility coefficients does not allow for any source of correlation, be it scale heterogeneity or a behavioral phenomenon. Moreover, the estimated distributions of ratios of coefficients (WTP values) can be biased if the correlations among coefficients are not captured [8].

Arguably, the paucity of studies using the RPL with correlated parameters lies in its computational burden, and the thorny interpretation of the model output, as we will discuss in the next section. Parallel to the growing use of RPL models, researchers have also embraced the use of hybrid choice models (HCMs) [9] to capture the effect that latent constructs such as attitudes, norms, perceptions, affects and beliefs have on choices [10]. Whilst this model is particularly suitable to obtain greater insights into attitudes as additional drivers of choice, its complexity may shy away less experienced practitioners.

When approaching the task of analyzing DCE data, a researcher may wonder which model to use. In this paper, we focus on the empirical comparison of three models that have been widely used in the applied DCE literature: the RPL with correlated parameters (RPL-C, henceforth), the RPL with uncorrelated parameters (RPL-UC, henceforth), and the HCM. What are the gains, and the losses, of using one model versus the others? Is employing these models justified from the theoretical and empirical point of view? Given the large number of current papers still using the RPL-UC, is this model still useful for policy recommendations? It is important to highlight that, in this paper, we do not consider discrete distributions of parameters leading to latent class type models that are able to accommodate inherent correlation.

We address these questions by applying the three models to a study of people’s preferences for the characteristics of an environmental program aimed at promoting renewable energy production through the growth of seaweed for anaerobic digestion. Our analysis is carried out with three different samples—England, Scotland and Northern Ireland—to explore the robustness of our results. We show that care should be placed when choosing which model to use, and the selection of the model should depend on both the outcome of statistical tests, as well as a priori expectations (i) of the importance of attitudinal variables in explaining the distribution of WTP, and (ii) covariances among the random parameters.

The paper is structured as follows: section 2 presents a review of studies using the RPL-UC, the RPL-C and the HCM; section 3 describes the econometric models; section 4 introduces the case study of the DCE on seaweed for energy production; section 5 presents the results of the econometric models, and section 6 concludes the paper with some recommendations on model selection.

## Literature review

The RPL is currently the most frequently used model to analyze stated choice data in environmental valuation, where the vast majority of the applications assume that the coefficients of the RPL are uncorrelated. From this vast literature, we can mention Agimass et al. [11], who study the preferences for different forest attributes using data from actual visits. With respect to the fisheries sector, Bronnmann and Asche [12] examine preferences for sustainable fisheries comparing ecolabelled wild salmon with farmed salmon, Ropars-Collet et al. [13] examine people’s preferences for the provision of amenities from the fishing sector along the coasts of the English Channel and the North Sea, and Paltriguera et al. [14] evaluate people’s preferences for recreational attributes of a coastal Marine Protected Area in England. Within conservation, Barrowclough and Alwang [15] study farmers’ attitudes towards conservation agriculture in Ecuador, Brouwer et al. [16] measure the non-market benefits of ecological river restoration, Fujino et al. [17] study attitudes towards a Japanese biodiversity conservation policy and Dechasa et al. [18] estimate the economic values of wetlands services. In a more general context, Huynh et al. [19] explore new ways of understanding and measuring preferences for different remediation technologies for contaminated environment approaches in New South Wales, Australia and Seeteram et al [20] analyze the willingness to pay for restoration of ecosystem services in south Florida (US).

Probably due to the computational complexity and non-trivial interpretation, the use of an RPL-C has been less frequently applied in the environmental valuation literature. Recent examples include Alberini et al. [21], who seek to estimate the benefits of climate change mitigation, as measured by the public’s willingness to pay (WTP) for such policies, Waldman et al. [22], who evaluate farmers’ preferences for perennial attributes of pigeon pea intercropped with maize in central and southern Malawi, Wakamatsu et al. [23], who value whale conservation using data collected from anti-whaling populations in Australia and Japan, and Mariel and Meyerhoff [24] who compare the outcomes of RPL models with correlated and uncorrelated parameters analyzing farmers’ willingness to accept compensation for implementing agri-environmental measures in Germany. Finally, Bjørnåvold et al. [25] analyze whether European policymakers are more likely to fund dominant incumbent technologies to decarbonize transport rather than novel technologies.

In HCMs, latent variables represent the characteristics of individuals, which are typically constructs, like attitudes [9]. These latent variables are introduced into the model as endogenous and explained by socio-demographic variables while, at the same time, act as explanatory variables for observed indicators. Older applications of this approach in the environmental field generally support the finding that HCMs provide greater insight into attitudes as additional drivers of choice [26–31]. Mariel and Meyerhoff [32] compare the results from a HCM and an RPL-UC model, concluding that the choice between them depends on the objective of the analysis. If disentangling preference heterogeneity is most important, the hybrid model seems to be the preferred alternative.

More recent applications have analyzed the WTP for forest management targeting non-use value ecosystem services [33], peatland restoration in Scotland [34], and ecologically sustainable products [35]. Important theoretical findings regarding the HCM show that the statistical benefits of the HCM are smaller than previously believed [10]. The most important criticism, however, is presented in Chorus and Kroesen [36]. This criticism is based on two ideas. The first is that in an HCM, a latent variable is partly endogenous, thus, precluding a causal inference. The second is that the cross-sectional nature of the latent variable does not allow for claims concerning changes in the variable at the individual level. To the best of our knowledge, this criticism has not been satisfactorily addressed in the literature thus far. Moreover, the ongoing discussion about the use of the HCM focuses also on the quality and representativeness of the latent attributes included in the model. For example, Bahamonde-Birke et al. [37] discuss the differences between attitudes and perceptions, and, Borriello and Rose [38] discuss the use of global versus localized attitudinal responses.

## Model specifications

In this paper, we compare the estimations of three models: the RPL-UC, the RPL-C and the HCM. All three are based on the random utility theory (RUM) [39], under which the utility function is defined as:

$$U_{nt,j}=\beta_{n}{}^{\prime }x_{nt,j}+\varepsilon_{nt,j},$$ (1)

where $\beta_{n}{}^{\prime }$ is a vector of individual taste coefficients for respondent *n*, *x*_*nt*,*j*_ are attribute levels for individual *n* choosing alternative *j* on choice occasion *t*, and *ε*_*nt*,*j*_ is the error term, which is independently and identically distributed as extreme value Type I. According to [39], it is assumed that person *n* will choose alternative *i* on choice occasion *t* such that *U*_*nt*,*i*_ > *U*_*nt*,*j*_, ∀*j* ≠ *i*.

For both RPL models, the vector of *β*_*n*_ can be further broken down to:

$$\beta_{n}=\beta +\Theta z_{n}+\Gamma \nu_{n},$$ (2)

where *β* is a vector of the mean values of the parameters, *z*_*n*_ is the vector of observed socio-demographic variables that affect the mean of the random parameter distribution, and Θ is its associated parameter matrix. The random unobserved heterogeneous preferences are represented in the vector of *v*_*n*_, which is characterized by:

$$E(v_{n})=0\;\text{and}\;Var(v_{n})=\sum =diag[\sigma_{1},\sigma_{2},\ldots \sigma_{K}].$$ (3)

We assume that the parameters *σ*_1_, *σ*_2_, …, *σ*_*K*_ are known constants and the corresponding lower triangular matrix Γ is to be estimated. This is where the RPL-UC model and the RPL-C model differ. In the former, the triangular matrix is actually a diagonal matrix Γ = *diag*[*γ*_1_, *γ*_2_, …, *γ*_*K*_], since we assume there are no correlations between the parameters. In the latter, the full variance-covariance matrix is:

$$\text{Var}\left(\beta_{n}\right)=\Gamma \Sigma \Gamma^{\prime }\text{.}$$ (4)

The corresponding probability function of person *n* choosing alternative *i* on choice occasion *t*, for both the RPL-UC and the RPL-C models, is given by:

$$P_{nt,i}=\frac{\text{exp}\left(x_{nt,i}^{\prime }\beta_{n}\right)}{\sum_{j=1}^{J}\text{exp}(x_{nt,j}^{\prime }\beta_{n})},$$ (5)

and the conditional probability of individual *n* making a series of choices for *T* choice occasions is given by:

$$P_{n}|\beta_{n}=\prod_{t=1}^{T}P_{nt,i}|\beta_{n}.$$ (6)

The vector *β*_*n*_ is unknown, therefore it is necessary to integrate it out of the probability. The log likelihood function of the conditional probability for the two RPL models is:

$$logL=\sum_{n=1}^{N}ln\int_{\beta_{n}}\prod_{t=1}^{T}P_{n}|\beta_{n}f\left(\beta ,\Omega \right)d\beta_{n},$$ (7)

where *f*(*β*, Ω) is the density function of the random variable *β*_*n*_, which depends on a set of parameters Ω. As there is no closed form solution, *logL* must be maximized by simulation for any given value Ω(Train, 2009) [40].

Both RPL models allow for heterogeneous preferences, that is, random taste variations between people. Thus, a distribution for each parameter has to be chosen; however, no consensus has been reached on how this should be done. Following a standard approach in the literature [41], we assume a log-normal distribution for the coefficient of the *cost* attribute, whose sign is reversed for the estimation process, and a normal distribution for the coefficients of other attributes.

McFadden and Train [7] have shown that any choice model, with any distribution of preferences, can be approximated to any degree of accuracy by a mixed logit. As the most widely used mixed logit derivation is based on random coefficients as presented above, the RPL model is one of the most frequently used models by discrete choice practitioners. The application of the RPL-UC model, however, imposes constraints on the model by assuming that the variance-covariance matrix of the parameters is diagonal. This issue is closely related to the debate concerning scale heterogeneity. Heterogeneity among respondents can appear either due to taste or scale heterogeneity, where one cannot be disentangled from the other due to the ever-present confounding issue [8, 42]. The RPL-C model is flexible since it allows for correlation among the parameters, thus preventing scale heterogeneity from being absorbed by the estimated taste parameters, which otherwise occurs when the scale is fixed in the RPL-UC model. Another direct implication is that the uncorrelated random coefficients in the utility function lead to a specific and restricted correlation structure of the WTP values [43]. That is why the RPL-C model compared to the RPL-UC model is a more flexible approach and the application of the RPL-UC model should always be justified by a proper statistical test.

In an HCM, *U*_*nt*,*j*_ also depends on the latent variable, *LV*_*n*_, and a vector of parameters *α* usually representing the interaction terms of the latent and explanatory variables. That is why Eq (2) is extended to

$$\beta_{n}=\beta +\Theta z_{n}+A\;LV_{n}+\Gamma \nu_{n}.$$ (8)

where *A* is a matrix of new parameters corresponding to the latent variables, *LV*_*n*_. For the sake of simplicity, we assume there is only one latent variable that is defined by a structural equation

$$LV_{n}=h\left(z_{n},\gamma \right)+\omega_{n},$$ (9)

where *h*(*z*_*n*_, *γ*) represents the determinist part of *LV*_*n*_ and the specification is *h*(⋅), which, in our case, is linear, with *z*_*n*_ being a vector of the same socio-demographic variables included in (8) and *γ* being a vector of parameters. Additionally, *ω*_*n*_ is a normally distributed random disturbance with zero mean and standard deviation *σ*_*ω*_. If there are more latent variables defined in (8) and (9) the error term *ω*_*n*_ becomes a vector with a multivariate distribution. Different restrictions implemented in (8) and (9), together with the distributional assumptions of the error terms *v*_*n*_ and *ω*_*n*_, lead to different identification and estimation strategies [10, 41]. In our paper, we include only one latent variable that represents the level of environmental friendliness of the respondents, selected through an exploratory factor analysis on a set of attitudinal questions. The HCM described above is only one possible setting of an HCM that is based on an RPL specification. The latent variable, *LV*_*n*_, can be incorporated into other parts of the utility function, for example, as a scale function [44].

The variance-covariance matrix of the utility parameters *β*_*n*_ in (8) of our HCM is assumed to be diagonal, that is, the parameters are assumed to be uncorrelated. The inclusion of correlated parameters would lead to 21 new parameters to be added to an already extremely high number of parameters (123) in the HCM. Thousands of individuals in the sample would be needed to estimate this kind of model with a certain level of confidence. As our samples include only hundreds of individuals, we opted for a restricted version of the model that allows for an RPL-UC, RPL-C and HCM comparison and it does not change the main message of our paper: given the limited sample sizes usually encountered in environmental valuation literature, the estimated distributions of the WTP can be very similar, but the model should be always chosen according to the underlying assumptions to be tested.

Measurement equations use the values of the attitudinal indicators as dependent variables, and explain their values through the latent variable. The *ℓ*^*th*^ indicator (of a total of *L* indicators) for respondent *n* is, therefore, defined as:

$$I_{ln}=m\left(LV_{n},\zeta \right)+v_{n},$$ (10)

where the indicator *I*_*ℓn*_ is a function of the latent variable *LV*_*n*_ and a vector of parameters *ζ*. The specification of *v*_*n*_ determines the behavior of the measurement model and depends on the nature of the indicator. Responses to the environmental statements in our case study are collected using a Likert-type response scale from 1 to 4, thus, the measurement equations are given by a typical ordinal logit [30].

For a discrete indicator with (in our case) 4 levels ***i***_1_, ***i***_2_, ***i***_3_, ***i***_4_, such that ***i***_1_ < ***i***_2_ < ***i***_3_ < ***i***_4_, the measurement equation for individual ***n*** is modelled as an ordered logit model for the latent variable, where ***τ***_*ℓ*,1_, ***τ***_*ℓ*,2_, and ***τ***_*ℓ*,3_, are thresholds that need to be estimated:

$$I_{ln}=\left\{\begin{array}{ll} i_{1} & if-\infty <LV_{n}\leq \tau_{l,1}\\ i_{2} & if\;\tau_{l,1}<LV_{n}\leq \tau_{l,2}\\ i_{3} & if\;\tau_{l,2}<LV_{n}\leq \tau_{l,3}\\ i_{4} & if\;\tau_{l,3}<LV_{n}<\infty \end{array}\right..$$

The likelihood of a specific observed value of *I*_*ℓn*_ (*ℓ* = 1, 2, …, 7) is then given by

$$L_{I_{ln}}=I_{\left(I_{ln}=i_{1}\right)}\left[\frac{\text{exp}\left(\tau_{l,i_{1}}-\zeta_{l}LV_{n}\right)}{1+\text{exp}\left(\tau_{l,i_{1}}-\zeta_{l}LV_{n}\right)}\right]+$$

$$\overset{3}{\underset{k=2}{\sum }}I_{\left(I_{ln}=i_{k}\right)}\left[\frac{\text{exp}\left(\tau_{l,k}-\zeta_{l}LV_{n}\right)}{1+\text{exp}\left(\tau_{l,k}-\zeta_{l}LV_{n}\right)}-\frac{\text{exp}\left(\tau_{l,\left(k-1\right)}-\zeta_{l}LV_{n}\right)}{1+\text{exp}\left(\tau_{l\left(k-1\right)}-\zeta_{l}LV_{n}\right)}\right]+$$

$$I_{\left(I_{ln}=i_{4}\right)}\left[1-\frac{\text{exp}\left(\tau_{l,3}-\zeta_{l}LV_{n}\right)}{1+\text{exp}\left(\tau_{l,3}-\zeta_{l}LV_{n}\right)}\right],$$

Where ***ζ***_***ℓ***_ measures the impact of the latent variable ***LV***_***n***_ on indicator ***I***_***ℓn***_ and ***τ***_**ℓ,1**_, ***τ***_**ℓ,2**_, ***τ***_**ℓ,3**_ are a set of estimated threshold parameters. In practice, each of ***τ***_**ℓ,1**_, ***τ***_**ℓ,2**_, ***τ***_**ℓ,3**_ are estimated using a set of auxiliary parameters ***δ***_**ℓ,1**_, ***δ***_**ℓ,2**_ such that

$$\tau_{l,2}=\tau_{l,1}+\delta_{l,1}$$

$$\tau_{l,3}=\tau_{l,2}+\delta_{l,2}$$

where *δ*_*ℓ*,1_, *δ*_*ℓ*,2_ ≥ 0, ∀ *ℓ*. The definition of the auxiliary parameters assures that *τ*_*ℓ*,1_ < *τ*_*ℓ*,2_ < *τ*_*ℓ*,3_ < *τ*_*ℓ*,4_.

Finally, the model is estimated by maximum simulated likelihood. The estimation involves maximizing the joint likelihood of the observed sequence of choices (*P*_*n*_) defined in (5) and the observed answers to the attitudinal questions ($L_{I_{ln}}$). The two components are conditional on the given realization of the latent variable *LV*_*n*_. Accordingly, the log-likelihood function of the model is given by integrating over *ω*_*n*_:

$$LL\left(\beta ,\gamma ,\zeta ,\tau \right)=\sum_{n=1}^{N}ln\int_{\omega }(P_{n}\prod_{l=1}^{7}L_{I_{ln}})g\left(\omega \right)d\omega .$$ (11)

Thus, the joint likelihood function (11) depends on the parameters of the utility functions included in (1), the parameters of the socio-demographic interactions in the latent variable specification defined in (9), and the parameters of the measurement equations defined in (10). Daly et al. [41] describe different identification procedures. We follow the Bolduc normalization by setting σ_ω_ equal to 1. All model components are estimated simultaneously using PythonBiogeme [45]. To compare the results of the three models, we study the differences in *median* WTP values, since in the case of non-symmetric WTP distributions, typically found in environmental valuation, they are more representative of the WTP distribution than the *mean* WTP values [24].

## Case study

We apply the RPL-UC, RPL-C, and the HCM to analyze people’s WTP to grow seaweed for biogas production, as part of a renewable energy program. After conducting focus groups and piloting, the DCE was conducted online from October 2017 to January 2018 among respondents from England, Scotland and Northern Ireland, who were recruited by a survey company. Each individual had to choose between three unlabeled alternatives, one of which was the status quo. Two of the alternatives were comprised of four attributes, as can be seen in Table 1: (i) *the number of households powered* using seaweed as a source of biogas; (ii) *the percentage of coastline used for seaweed farms*; (iii) *an increase in the electricity bill per year*, or the cost; and (iv) *perks*, which were low-cost nudges designed to encourage respondents to opt in. The third alternative simply said “No change from your current situation”.

**Table 1:** Attributes and their levels.

Attributes	Levels
Number of households powered	45,000; 85,000; 130,000
Percentage of coastline used for seaweed farms	10%, 20%, 30%
Increase in electricity bill per year	£10, £20, £50, £100, £150
Perks	Facebook profile picture overlay, A letter with your contribution, Nothing

**Fig 1:**
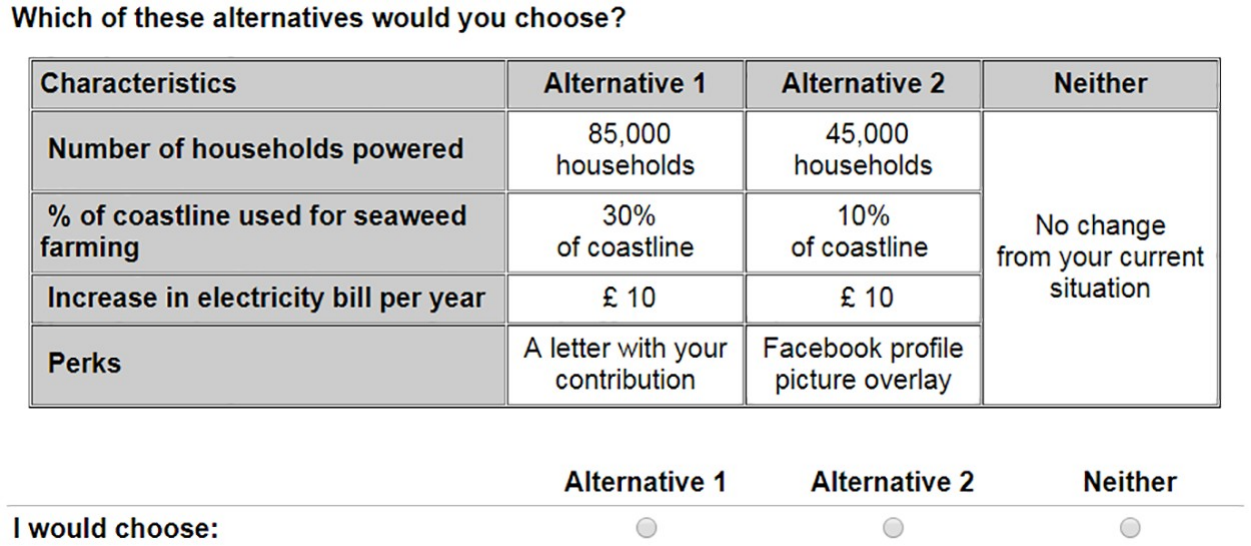
Sample choice occasion.

Using Ngene, a D-efficient design for a random parameter model was generated, with three blocks, 10 rows in each one [46]. We assumed normally (*N*) distributed Bayesian priors, with uniformly (*U*) distributed standard deviations, during the generation of the experimental design. The assumed distributions for the coefficients of the dummy coded attributes *the number of households powered* and *perks* were *N*(0.2,0.1) for the mean and *U*(0.1,0.3) for the standard deviation. We substituted *N*(0.2,0.1) by *N*(−0.2,0.1) in the case of the *percentage of coastline* as this attribute was expected to cause disutility. The Bayesian prior for the coefficient of the *increase in the electricity bill per year* was assumed *N*(−0.1,0.03) distributed. Respondents were presented with 10 choice occasions, such as in Fig 1, in a randomized order.

Before the DCE, individuals answered “warm-up” questions, taken from two Eurobarometer questionnaires, about their attitudes on climate change [47] and on the environment [48]. These questions were selected from a long list of questions from the Eurobarometer surveys through focus groups discussions. A subset of these questions was used to capture the latent attitude for the HCM representing a pro-environmental attitude. The exact wording of these questions, along with the direction of the association with the latent variable construct pro-environmentalism, can be seen in Table 2.

**Table 2:** Attitudinal questions.

		In your opinion, to what extent do the following factors influence your quality of life?	Codification of answers
*env1*	-	State of the environment	1 = very much, 4 = not at all
*env2*	-	Economic factors	1 = very much, 4 = not at all
*env3*	-	Social factors	1 = very much, 4 = not at all
*env4*	-	How important is protecting the environment to you personally?	1 = very important, 4 = not at all important
*env5*	-	You are willing to buy environmentally friendly products even if they cost a little bit more.	1 = totally agree, 4 = totally disagree
*env6*	-	As an individual you can play a role in protecting the environment in your country.	1 = totally agree, 4 = totally disagree
*env7*	-	The big polluters should be mainly responsible for making good the environmental damage they cause.	1 = totally agree, 4 = totally disagree

Pro-environmental respondents are expected to be willing to pay more for the environmentally-friendly program. The low-cost nudges are not anticipated to have a positive effect on pro-environmental individuals since an external reward is probably unnecessary for these respondents. The importance of the remaining two attributes to environmentally-friendly respondents is unclear. On the one hand, powering more households with renewable energy means reducing nonrenewable energy consumption. On the other hand, the size of the project itself might not matter to them, but rather their participation in it. Lastly, the percentage of the coastline used for farming seaweed could be seen in a positive light since it is being used for renewable energy; however, it could also be viewed as altering an eco-system, thus generating a negative effect.

Demel et al [49] analyze the same dataset using the simpler RPL-UC model without any interactions with the socio-demographic variables and not using responses to any attitudinal questions, finding that people are willing to use more coastline to farm seaweed in order to power more households. That is, they are willing to make a trade-off between the visual disamenity caused by the seaweed farms and producing more green energy. We extend the results of this work in three different directions. Firstly, we include interactions of the socio-demographic variables with the random utility coefficients in the RPL-UC, RPL-C and HCM to disentangle the observed preference heterogeneity. Secondly, we incorporate the responses to the attitudinal questions representing the pro-environmental orientation into the HCM. These responses should disentangle the observed preference heterogeneity even more. Finally, we compare the results of three different approaches in order to analyze their precision.

## Results

### Descriptive statistics

Table 3 lists the socio-demographic variables used in this study, along with the corresponding mean, median, standard deviation and the range, for each of the variables. 1097 respondents completed the choice experiment for England, 537 for Scotland, and 390 for Northern Ireland; 424 respondents had to be dropped because they were outliers, did not answer the socio-demographic or environmental attitude questions or were protesters, thus, the final sample sizes were 868, 424, and 308 respondents for England, Scotland and Northern Ireland, respectively. Table 3 reports descriptive statistics for the clean samples. As can be seen from the upper half of the table, the three continuous variables do not differ much for the three countries.

**Table 3:** Descriptive statistics.

*Continuous Variables*	Mean	Median	St. Dev.	Min.	Max.
**England**					
Age	40.1	39	12.9	18	65
Number of children	0.8	0	1.1	0	5
Left-right ideology*	5.2	5	2.0	1	10
**Scotland**					
Age	40.4	40	12.9	18	65
Number of children	0.8	0	1.0	0	5
Left-right ideology	4.8	5	1.7	1	10
**Northern Ireland**					
Age	40.5	40	12.9	18	65
Number of children	1.0	1	1.2	0	7
Left-right ideology	5.2	5	2.0	1	10
*Dummy Variables*		**England**	**Scotland**	**Northern Ireland**	
Female		50.6%	49.3%	50.0%	
Cohabitating partnership		62.2%	61.1%	58.8%	
Employed		71.7%	71.2%	69.8%	
High education		52.6%	54.7%	54.9%	
High income		24.0%	24.1%	25.0%	
Regularly buy “green” energy		17.5%	14.9%	16.2%	

*1 = left, 10 = right.

The socio-demographic variables, which are dummy variables, are listed in the lower half of Table 3. A *cohabitating partnership* was defined as including married or cohabitating respondents; *employed* includes respondents employed full-time, part-time, as well as those who are self-employed. A *high education* is considered anything from a Foundation degree or higher (Foundation degree, Bachelor’s, Master’s, PhD), while a *high income* was characterized as having a gross annual household income of £48,001 or greater. Lastly, *regularly buy “green” energy* refers to if the respondent, or anyone in his or her household, regularly buys green energy. The proportions of these groups do not differ greatly between the countries.

Finally, the samples are representative with respect to age and gender of the general population in each of the three countries. The 2011 census results [50–52] confirm this with the mean ages for England, Scotland and Northern Ireland as 39.3, 40.3 and 37.6, respectively. Furthermore, approximately half of the population for each country is female (50.8% for England, 51.5% for Scotland and 51% for Northern Ireland).

Respondents were also asked a series of questions about their attitudes on the environment and climate change, as can be seen in Table 2. We excluded participants who answered “I don’t know” to these questions, approximately 2% of respondents for most questions, and slightly higher for some questions (the highest was 4.8% for one question). The breakdown of the answers to those questions is displayed in Table 4, by country. Values closer to 1 represent a pro-environmental attitude. The distribution of the responses is similar between the three countries. Furthermore, more than half of the respondents display a pro-environmental attitude (responses 1 and 2).

**Table 4:** Responses to the attitudinal questions.

	1	2	3	4
**England**				
*env1*	24.4%	48.5%	25.0%	2.1%
*env2*	32.5%	49.7%	16.9%	0.9%
*env3*	24.9%	48.7%	24.0%	2.4%
*env4*	49.1%	45.9%	4.6%	0.5%
*env5*	21.7%	56.1%	19.5%	2.8%
*env6*	43.5%	48.0%	7.7%	0.7%
*env7*	59.3%	35.6%	4.4%	0.7%
**Scotland**				
*env1*	24.8%	49.3%	24.1%	1.9%
*env2*	36.3%	48.3%	13.9%	1.4%
*env3*	24.5%	52.4%	21.5%	1.7%
*env4*	47.9%	46.0%	5.7%	0.5%
*env5*	21.7%	56.1%	18.9%	3.3%
*env6*	42.7%	49.1%	8.0%	0.2%
*env7*	56.4%	38.9%	4.5%	0.2%
**Northern Ireland**				
*env1*	22.4%	51.0%	22.1%	4.5%
*env2*	34.7%	50.0%	13.0%	2.3%
*env3*	23.1%	51.3%	21.1%	4.5%
*env4*	43.2%	50.0%	5.8%	1.0%
*env5*	18.2%	59.4%	20.1%	2.3%
*env6*	44.8%	49.0%	4.2%	1.9%
*env7*	58.1%	36.7%	4.2%	1.0%

Note: 1 = most environmentally-friendly answer, 4 = least environmentally-friendly answer. Specifically, for env1-env3, 1 = very much, 4 = not at all; for env4, 1 = very important, 4 = not at all important; for env5-env7, 1 = totally agree, 4 = totally disagree.

### Principal component analysis

The set of seven attitudinal questions listed in Table 2 are used to capture one latent construct related to the pro- and anti-environmental behavior. This can be confirmed by an exploratory factor analysis. After this check, the latent construct can become a part of the HCM by the means of the attitudinal questions. The Kaiser, Meyer, Olkin measure of sampling adequacy (MSA) suggests that the data seems appropriate for principal component analysis for all three countries (England: Overall MSA = 0.78; Scotland: Overall MSA = 0.77; Northern Ireland: Overall MSA = 0.75). Moreover, Bartlett’s test of sphericity generates *p-values* < 0.01, indicating that the data are likely to be suitable for principal components analysis.

As can be seen in Table 5, the principal components extraction produced two factors with eigenvalues greater than 1.0, for each of the three countries. The first factor explained 39.6%-42.1% of the total variance in the seven items, depending on the country. The second factor explained an additional 16.5%-17.9% of the total variation, a sharp decrease from the first.

**Table 5:** Exploratory factor analysis.

	Eigenvalues and percentages			Factor loadings		
Factor	Eigenvalue	Percentage	Cumulative	Variable	Factor1	Factor2
**England**						
*Factor1*	2.77	39.6%	39.6%	*env1*	0.45	0.14
*Factor2*	1.15	16.5%	56.0%	*env2*	0.37	0.53
*Factor3*	0.94	13.5%	69.5%	*env3*	0.38	0.53
*Factor4*	0.65	9.3%	78.8%	*env4*	0.42	-0.35
*Factor5*	0.54	7.7%	86.5%	*env5*	0.40	-0.40
*Factor6*	0.48	6.9%	93.4%	*env6*	0.37	-0.35
*Factor7*	0.46	6.6%	100.0%	*env7*	0.21	-0.11
**Scotland**						
*Factor1*	2.95	42.1%	42.1%	*env1*	0.45	0.18
*Factor2*	1.25	17.9%	60.0%	*env2*	0.38	0.49
*Factor3*	0.91	13.0%	73.0%	*env3*	0.36	0.55
*Factor4*	0.56	8.0%	81.0%	*env4*	0.42	-0.35
*Factor5*	0.52	7.4%	88.4%	*env5*	0.39	-0.34
*Factor6*	0.43	6.2%	94.5%	*env6*	0.38	-0.37
*Factor7*	0.38	5.5%	100.0%	*env7*	0.24	-0.21
**Northern Ireland**						
*Factor1*	2.82	40.3%	40.3%	*env1*	0.49	0.05
*Factor2*	1.23	17.6%	57.9%	*env2*	0.40	-0.49
*Factor3*	0.97	13.9%	71.7%	*env3*	0.34	-0.56
*Factor4*	0.67	9.6%	81.3%	*env4*	0.40	0.34
*Factor5*	0.50	7.2%	88.5%	*env5*	0.39	0.46
*Factor6*	0.44	6.2%	94.7%	*env6*	0.37	0.26
*Factor7*	0.37	5.2%	100.0%	*env7*	0.18	-0.24

All of the factor loadings for the first factor were positive and moderately strong. The second factor had some factor loadings that were positive and others that were negative, inconsistent between the three countries. The all positive factor loadings in the first factor are in line with capturing an environmentally-friendly (or an environmentally-unfriendly) attitude, since an environmentally-(un)friendly person would tend to answer each of the seven questions in the same direction. The inconsistent factor loadings for the second factor do not make sense conceptually, and it is unclear what kind of attitudes would be represented by this second factor. Thus, it was decided that only the first factor would be used in the HCM, to represent the degree of a pro-environmental attitude held by the respondent. Lastly, a reliability analysis was conducted to verify the internal consistency of the seven questions. The findings revealed that it formed a reliable scale for each of the three countries (England: Cronbach’s α = 0.74; Scotland: Cronbach’s α = 0.77; Northern Ireland: Cronbach’s α = 0.74).

### Model results

As described in the model specifications section, the HCM includes a latent variable, which in our case is defined as having a pro-environmental attitude, characterized according to (9) by the socio-demographic variables listed in Table 3. For example, people with more children might have a more environmentally-friendly attitude since they, most likely, want their children to have a good future. To make the three models similar and easily comparable, the same interactions with socio-demographic variables *z*_*n*_ are included in the utility function in (2). As recommended by Vij and Walker [10], in the HCM, apart from the utility function, the same socio-demographic variables are also included in (9).

The main estimates of the RPL-UC model, RPL-C model and the HCM for England, Scotland and Northern Ireland are presented in Tables 6–8.

**Table 6:** Model estimations for England.

	Uncorrelated RPL	Correlated RPL	Hybrid Choice Model
*Alternative specific constants*			
ASC1	0.072* (0.038)	0.017 (0.037)	0.072* (0.038)
Status Quo	-3.095*** (0.125)	-3.341*** (0.094)	-3.076*** (0.125)
*Attributes (means)*			
Cost	-4.392*** (0.239)	-4.414*** (0.229)	-4.331*** (0.239)
85,000 households powered *(baseline = 45*,*000 households)*	0.808*** (0.271)	0.983*** (0.319)	0.825*** (0.270)
130,000 households powered *(baseline = 45*,*000 households)*	0.852** (0.402)	1.252*** (0.454)	0.807** (0.399)
20% of coastline used *(baseline = 10%)*	0.170 (0.294)	0.095 (0.295)	0.158 (0.293)
30% of coastline used *(baseline = 10%)*	-0.127 (0.327)	-0.129 (0.364)	-0.133 (0.327)
Letter with contribution perk	0.221 (0.277)	0.440 (0.292)	0.227 (0.271)
Facebook profile picture perk	-0.447 (0.290)	-0.009 (0.327)	-0.469 (0.288)
*Attributes (standard deviations)*			
Cost	1.119*** (0.036)	1.128*** (0.045)	1.042*** (0.041)
85,000 households powered	0.397*** (0.136)	1.055*** (0.084)	0.346** (0.147)
130,000 households powered	1.267*** (0.105)	1.947*** (0.114)	1.228*** (0.102)
20% of coastline used	0.813*** (0.096)	1.010*** (0.085)	0.768*** (0.102)
30% of coastline used	1.183*** (0.092)	1.433*** (0.093)	1.190*** (0.094)
Letter with contribution perk	0.358** (0.139)	0.820*** (0.096)	0.387*** (0.122)
Facebook profile picture perk	0.603*** (0.097)	0.954*** (0.101)	0.574*** (0.099)
Number of observations	8680	8680	8680
Number of respondents	868	868	868
Number of estimated parameters	79	100	123
Log-likelihood	-6442.9	-6255.5	-11969.5
AIC	13043.8	12711.0	24185.0
BIC	13602.2	13417.9	25054.5
McFadden’s Pseudo R-squared	0.30	0.32	0.19

Note:

***, **, * denote significance at the 1%, 5%, and 10% level, respectively.

**Table 7:** Model estimations for Scotland.

	Uncorrelated RPL	Correlated RPL	Hybrid Choice Model
*Alternative specific constants*			
ASC1	-0.031 (0.057)	-0.068 (0.053)	-0.037 (0.057)
Status Quo	-3.253*** (0.175)	-3.487*** (0.142)	-3.254*** (0.176)
*Attributes (means)*			
Cost	-3.685*** (0.363)	-4.113*** (0.331)	-3.537*** (0.526)
85,000 households powered *(baseline = 45*,*000 households)*	1.404*** (0.378)	0.986** (0.425)	1.402*** (0.378)
130,000 households powered *(baseline = 45*,*000 households)*	1.749*** (0.614)	1.152* (0.598)	1.774*** (0.619)
20% of coastline used *(baseline = 10%)*	-0.002 (0.368)	0.001 (0.409)	-0.015 (0.370)
30% of coastline used *(baseline = 10%)*	0.226 (0.548)	0.175 (0.557)	0.131 (0.559)
Letter with contribution perk	0.171 (0.385)	0.165 (0.424)	0.190 (0.384)
Facebook profile picture perk	-0.354 (0.455)	-0.259 (0.505)	-0.327 (0.457)
*Attributes (standard deviations)*			
Cost	1.159*** (0.057)	1.118*** (0.063)	1.125*** (0.096)
85,000 households powered	0.056 (0.107)	0.756*** (0.116)	0.127 (0.210)
130,000 households powered	1.153*** (0.130)	1.656*** (0.157)	1.133*** (0.137)
20% of coastline used	0.647*** (0.126)	0.872*** (0.165)	0.618*** (0.131)
30% of coastline used	1.305*** (0.135)	1.610*** (0.144)	1.307*** (0.147)
Letter with contribution perk	0.322 (0.213)	0.824*** (0.149)	0.302 (0.187)
Facebook profile picture perk	0.772*** (0.139)	1.139*** (0.145)	0.769*** (0.129)
Number of observations	4240	4240	4240
Number of respondents	424	424	424
Number of estimated parameters	79	100	123
Log-likelihood	-3106.1	-3016.6	-5746.4
AIC	6370.2	6233.2	11738.8
BIC	6872.0	6868.4	12520.1
McFadden’s Pseudo R-squared	0.31	0.33	0.20

Note:

***, **, * denote significance at the 1%, 5%, and 10% level, respectively.

**Table 8:** Model estimations for Northern Ireland.

	Uncorrelated RPL	Correlated RPL	Hybrid Choice Model
*Alternative specific constants*			
ASC1	-0.047 (0.058)	-0.110* (0.059)	-0.046 (0.057)
Status Quo	-2.984*** (0.195)	-3.322*** (0.149)	-3.010*** (0.194)
*Attributes (means)*			
Cost	-4.557*** (0.354)	-4.486*** (0.309)	-4.695*** (0.365)
85,000 households powered *(baseline = 45*,*000 households)*	0.431 (0.422)	0.908* (0.509)	0.421 (0.404)
130,000 households powered *(baseline = 45*,*000 households)*	0.320 (0.625)	0.919 (0.714)	0.305 (0.613)
20% of coastline used *(baseline = 10%)*	0.034 (0.357)	0.314 (0.442)	0.023 (0.347)
30% of coastline used *(baseline = 10%)*	0.212 (0.401)	-0.070 (0.514)	0.252 (0.398)
Letter with contribution perk	0.405 (0.459)	0.374 (0.506)	0.423 (0.437)
Facebook profile picture perk	-0.402 (0.496)	-0.402 (0.578)	-0.315 (0.481)
*Attributes (standard deviations)*			
Cost	1.039*** (0.056)	1.041*** (0.061)	0.997*** (0.064)
85,000 households powered	0.080 (0.313)	1.007*** (0.137)	0.119 (0.183)
130,000 households powered	1.092*** (0.144)	1.814*** (0.183)	0.989*** (0.143)
20% of coastline used	0.521*** (0.175)	0.759*** (0.134)	0.437** (0.178)
30% of coastline used	0.681*** (0.154)	1.034*** (0.151)	0.685*** (0.158)
Letter with contribution perk	0.493*** (0.163)	0.935*** (0.130)	0.300 (0.507)
Facebook profile picture perk	0.723*** (0.137)	1.218*** (0.146)	0.665*** (0.135)
Number of observations	3080	3080	3080
Number of respondents	308	308	308
Number of estimated parameters	79	100	123
Log-likelihood	-2348.6	-2282.3	-4298.5
AIC	4855.2	4764.6	8843.0
BIC	5331.8	5367.9	9585.0
McFadden’s Pseudo R-squared	0.28	0.30	0.18

Note:

***, **, * denote significance at the 1%, 5%, and 10% level, respectively.

For our purposes of model comparison, only the alternative-specific constants (ASC), estimated coefficients of each attribute, and the standard deviations of each attribute, along with the corresponding standard errors in brackets below, are presented in these tables. The remaining estimated coefficients corresponding to each of the three models, such as the various mean-shifters and structural equations, can be found in S1 Tables A1-A3 in S1 File.

The standard deviations for the RPL-C models were calculated using the lower triangular matrix, Γ, listed in S1 Tables A1-A3 in S1 File. The models were estimated using PythonBiogeme [45] and the gmnl package in R [53] using 2000 Halton draws.

Comparing the RPL-UC and the RPL-C models, the McFadden’s Pseudo R-squared is the highest in the RPL-C model for all three countries, indicating a better fit. Similarly, the AIC and BIC values are lower in the RPL-C model than in the RPL-UC model, except for Northern Ireland. The McFadden’s Pseudo R-squared of the HCM is not directly comparable to the RPL-UC and RPL-C figures because, apart from the choice model part defined by (1), the HCM includes the structural Eq (9) and the measurement Eq (10). It also impacts the value of the log-likelihood, which influences the AIC and BIC values.

It is important to mention that the use of the RPL-UC should be properly justified. The null hypothesis *H*_0_: *Γ in* (4) *is diagonal* can be tested with an LR test against the alternative hypothesis *H*_*a*_: *Γ in* (4) *is not a diagonal matrix*. The degrees of freedom of this test are the number of elements below the main diagonal *Γ*, in our case 21 (6+5+4+3+2+1), or simply, the difference between the number of parameters estimated in the RPL-UC and the RPL-C (100–79). Taking into account the extremely large *Likelihood-ratio test statistics* presented in Table 9 for all three countries, caused by a large improvement in fit when comparing the RPL-C to the RPL-UC, the *p-values* indicate a rejection of the null hypothesis at any conventional level (the critical value is $\chi_{21}^{2}=32.67$). This means that the RPL-UC imposes incorrect restrictions that can lead to biased estimates. There are, therefore, non-zero correlations between the random utility coefficients caused by a behavioral phenomenon or phenomena, or by scale heterogeneity. We keep considering the RPL-UC in the analysis below to see how using an RPL-UC that has not been justified can affect the estimation of the parameters and WTP distributions.

**Table 9:** Likelihood-ratio test.

*Country*	*Likelihood-ratio test statistic*	*p-value*
England	374.8	<0.01
Scotland	179.0	<0.01
Northern Ireland	132.6	<0.01

There are some similarities between the estimates of the three models, but also some differences. The ASC representing the status quo option is negative and statistically significant at the 1% significance level for all three models and for all three countries. This indicates that respondents chose the status quo alternative significantly less than the other two, demonstrating that people prefer to opt into one of the seaweed programs rather than not.

All of the coefficients are assumed to have a normal distribution, except for *cost*, which is assumed to be log-normally distributed and its sign has been reversed for the estimation. In all three models, across all countries, the estimated coefficient of *cost* is negative and statistically significant at the 1% significance level. Since it is log-normally distributed, an estimated coefficient of -4.392 in the RPL-UC model for England corresponds to a median of −exp(−4.392) = −0.012, and a mean of $-\text{exp}\left(-4.392+\frac{0.239^{2}}{2}\right)=-0.013$. The means and medians are negative, as would be expected, since a higher cost decreases one’s utility.

Focusing on the estimation of the means of the distributions corresponding to the non-cost attributes, the RPL-UC and RPL-C models show very similar results in all three countries. The majority of these means are not significant at the 5% significance level, but their interpretation is related to the interaction coefficients presented in S1 Tables A1-A3 in S1 File. For example, the estimated mean for the attribute 85,000 households powered for England is 0.808; however, this corresponds to a non-interpretable situation, where all socio-demographic variables included in *z*_*n*_ in (2) are set to zero, which means even age is set to zero. The estimated mean for an individual who is 30 years old and is characterized by the other socio-demographic variables set to zero is, according to Table 6 and S1 Table A1 in S1 File, defined as 0.808 + 30 ⋅ 0.012 = 1.168. Therefore, each subgroup of the population characterized by a combination of possible values of the socio-demographic variables has its own mean value.

Regarding the HCM, the positive *ζ* values in the estimated measurement equations presented in S1 Table A1-A3 in S1 File indicate that, the higher value of the indicator (higher values are associated with anti-environmental behavior), the higher value of the latent variable. As can be seen in S1 Table A1-A3 in S1 File, the interaction effects of the latent variable with the non-cost attributes are not statistically significant in any of the three samples, except for the two perk attributes in Northern Ireland.

The interaction effect with cost is positive and statistically significant at the 1%, 10% and 5% significance level for England, Scotland and Northern Ireland, respectively. This means that, in all three countries, people with a more positive latent variable (less environmentally-friendly) are willing to pay less for the seaweed program. Since *cost* is log-normally distributed with a negative sign, a greater negative coefficient means a higher cost coefficient in absolute values, and, therefore, a lower WTP value.

Regarding the estimations of the structural equations involving the socio-demographic variables of the three HCMs presented in S1 Table A1-A3 in S1 File, various socio-demographic variables are statistically significant. The only socio-demographic variable statistically significant at the 1% level in all three analyzed countries is whether they buy “green” energy regularly. The negative coefficient for regularly buying “green” energy indicates that those people have a more negative value of the latent variable, which corresponds with more environmentally-friendly views. For the Scottish sample, the positive and statistical significance of political orientation means that the further right a respondent self-reports being, the less environmentally friendly they are. In the English sample, respondents who are more educated and have more children are more environmentally-friendly. These results are as expected.

### WTP results

**Fig 2:**
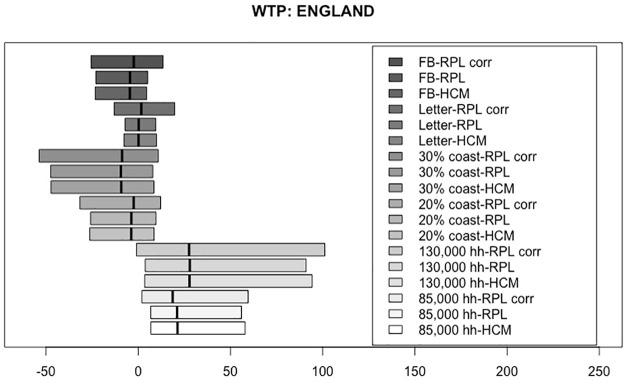
Distribution of WTP measures for England.

**Fig 3:**
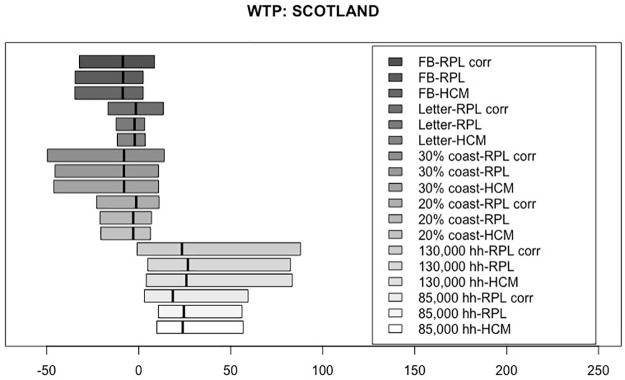
Distribution of WTP measures for Scotland.

**Fig 4:**
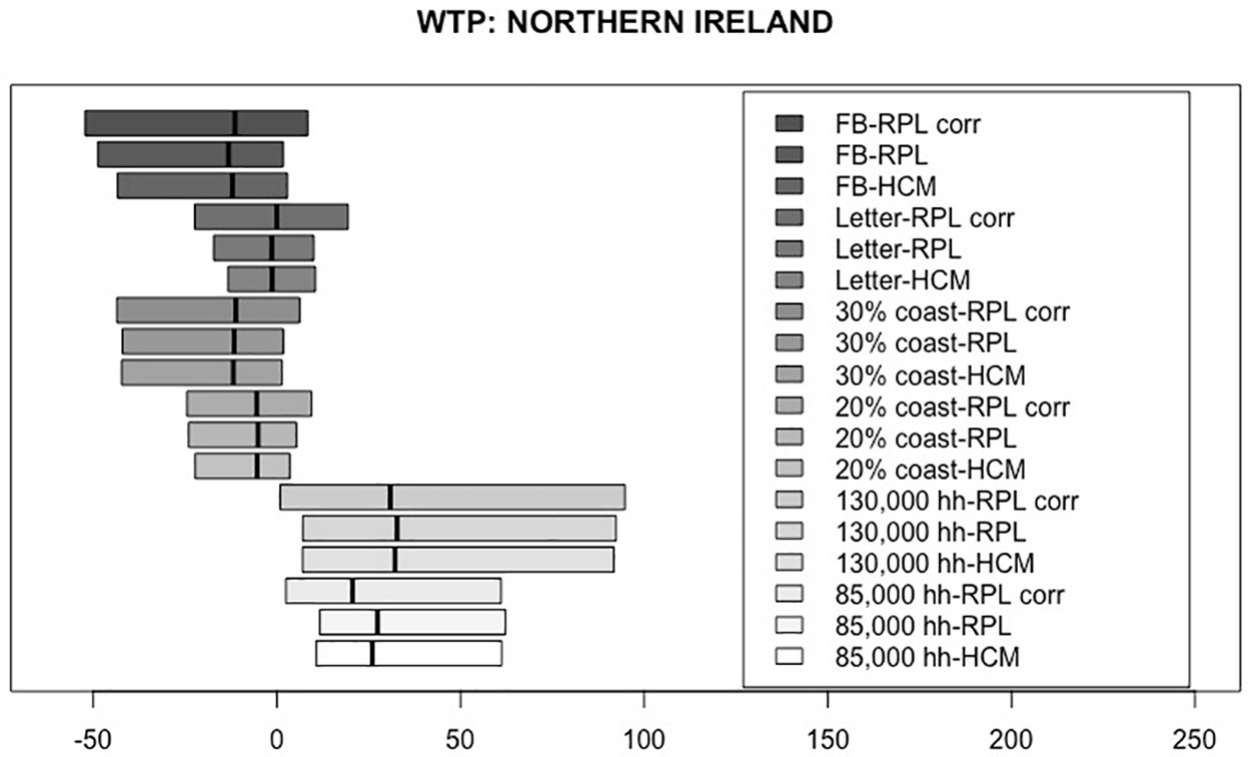
Distribution of WTP measures for Northern Ireland.

Based on the estimated coefficients in Tables 6–8, WTP measures were simulated corresponding to each of the three models for each country, given in pounds (£) per year. For the HCM, first, the latent variable was simulated using the socio-demographic values of our samples, as defined in (9), and then it was incorporated into the simulation of the WTP. Figs 2–4 present the WTP measures for each of the three models, by country. The boxplots range from the 25^th^ percentile to the 75^th^ percentile, with a vertical line marking the median. The WTP values for each of the attributes are displayed for the RPL-C model, the RPL-UC model and the HCM, moving from top to bottom.

Figs 2–4 show very similar figures for the three countries. Generally, the attributes with positive median WTP values are the number of households powered (85,000 households powered and 130,000 households powered). The boxplots representing 20% and 30% of the coastline used to farm seaweed are negative across all three countries, meaning that they are increasingly less desirable than the smallest value of 10%. As expected, the median values for the 30% coastline boxplot are greater in absolute value than those for 20% showing stronger aversion to using 30% of the coastline than 20%.

The median values of the boxplots representing the first perk, the letter, are close to zero. This means that, using the letter as a way to encourage more people to opt into a seaweed project seems to be ineffective. Moving on to the second perk, the Facebook profile picture overlay with an eco-friendly stamp, the median value for all three countries is negative and the corresponding distributions have much larger dispersion than the first perk. This means that people generally dislike the idea of using a Facebook profile picture overlay as an incentive to opt-in and that their preference heterogeneity is bigger than for the first perk, the letter.

Focusing on the model comparison of the three median values for each of the attributes presented in Figs 2–4, we find that they are very consistent across the three analyzed models. Furthermore, the three models present practically the same spread of the WTP distributions. A general result regarding this issue is difficult to establish, as the spread of the distributions depend on the underlying assumptions related to the distributions of the random coefficients. Nevertheless, what seems to be a repeated pattern in all datasets is that, generally, the widest distributions belong to the RPL-C. In spite of the fact that we used three different country samples for our estimations, and our findings seem to be relatively robust, the spread difference is so small that it is impossible to draw any general conclusion.

## Conclusions

The aim of this paper was to compare empirically three widely used models applied to three datasets devoted to renewable energy from seaweed. The relevant question is not which of these models we should prefer, but what our a priori assumptions and hypotheses should be, and then which model represents them correctly.

The RPL-C and HCM are very flexible models from a theoretical point of view. The large number of parameters, the need for a big sample, and the high computational burden of the estimation procedure are their clear drawbacks. For an inexperienced practitioner, the use of the RPL-C or the HCM can lead to a wrong application of the estimation procedure and an incomplete post-estimation analysis. For example, one of the simplest checks (not frequently done by many practitioners) of the model results based on simulated log-likelihood functions (as in our case) is the re-estimation of the same model with different sets of starting values. Models with such a high number of parameters can easily suffer from local maxima and flat regions of the log-likelihood function and the use of different starting values can, at least partially, cope with this problem. Many practitioners usually do not perform this simple post-estimation check because the estimation of models with such a high number of parameters is time consuming.

The RPL-UC model includes restrictions on the variance-covariance matrix of the random parameters that should be tested to justify the use of this model. These restrictions have been rejected in our case, and the application of the RPL-UC model is, therefore, inadequate. When we compared the output of this model with the other two more complex models, due to the limitation in sample size of our application, we found no differences in the distributions of the WTP values. Other applications may find differences between the models with similar sample size as ours. Therefore, this does not mean that the RPL-UC will always lead to the same outcome as the outcome from the RPL-C.

Practitioners should not stick with the widely used RPL-UC that leads to much simpler and faster estimation and easier interpretation than the RPL-C case and should test whether the triangular matrix of the variance-covariance matrix of the RPL-UC is diagonal. The software available for the estimation of the RPL-C model has been available for a long time. Some examples are Biogeme [54], the gmnl package in R [53], the Apollo package in R [55], Stata modules [56] and MatLab codes [57].

The hybrid model is based on the assumption that there are some latent attitudes that affect the respondent’s choices, and that these attitudes can be captured by a set of attitudinal questions. This allows for an interesting interpretation and, usually, for a deeper insight into the decision process. The latent attitude is usually captured by an ad-hoc or a standard scale previously proposed and tested in the literature, such as the New Environmental Paradigm, a scale defined for measuring a general attitude towards the environment [58]. In any case, a standardized set of questions is always recommended, and a preliminary multivariate analysis applied to the responses to the attitudinal questions should confirm that the questions do in fact represent the latent attitude. However, future research, not only in environmental valuation literature, should focus on how to improve the representation of the latent attitudes that influence people’s choices, such as global versus localized attitudes or perceptions [37, 38]. However, given the theoretical complexity of the HCM and the computational burden related to its estimation, this model should probably be applied by experienced practitioners only.

Whether or not one or more latent attitudes really affect respondent’s choices seems to be the key issue in the application of the HCM. That is, are the latent variables *LV*_*n*_ in (8) relevant variables for the *U*_*nt*,*j*_ in (1)? If they are, their omission leads to inconsistent estimates similar to the well-known case of omission of a relevant variable in a linear regression model. Therefore, if the utilities *U*_*nt*,*j*_ do depend on latent constructs, there is an omission of a relevant variable(s) issue in the RPL-C (and RPL-UC) that, similar to its effect in a classical linear regression model, biases the estimates. Interestingly, the HCM literature shows that the influence of these latent constructs on the utilities measured by the responses to the attitudinal questions is usually very low (the corresponding coefficients are usually small and frequently non-significant). One possible reason can be the use of inappropriate measures of these constructs [37, 38], and this is why this issue should be an important focus of future research in this field. In conclusion, if the preferences regarding the environmental good or service under study can be affected by specific individual attitudes, their measures should be collected and their appropriateness carefully tested during the preparation of the survey. In such a case it may be expected to find significant coefficients for the attitudinal variables in the HCM.

There is another issues related to the interpretation of the HCM outcome. It is highly case dependent whether any additional insight provided by the HCM is valuable enough for policymakers to warrant the implementation of this theoretically and empirically complex model. For example, in our case study, the inclusion of our latent variable in the HCM model has led to the conclusion that less environmentally-friendly people (the larger the value of the latent variable) are less willing to pay for the seaweed program. How to identify this sub-population to apply a specific environmental policy remains unanswered by the HCM because the structural equation that relates the latent variable to the socio-demographic variables in our study explains only a small part of the variation of the latent variable—as often found in the literature—because the latent variable is, and should be, truly latent.

Our DCE study is a fairly standard DCE: it has three attributes with three levels each, one attribute with five levels, two unlabeled alternative options and the status quo, with respondents facing ten DCE questions. The sample size is also quite comparable to many DCE studies, as our three samples comprise of 868 respondents in England, 424 in Scotland, and 308 in Northern Ireland. We have not found large differences across the models in terms of WTP distributions, but this result cannot be used as a justification for the application of a simpler model. In our DCE application, it is likely that a larger sample would have shown these differences, but in other applications, these differences can appear with much smaller samples.

In conclusion, we would suggest practitioners to estimate not only the RPL-UC, but also the RPL-C, given that the routines for the RPL-C are widely available in both commercial and free software. The RPL-C should not be estimated only if there are strong expectations that the triangular matrix of the variance-covariance matrix of the RPL-UC is diagonal. Finally, we would encourage using the HCM if researchers expect that attitudinal variables should play an important role in explaining the distribution of WTP, with great care to be placed not only in the execution of the model, but also in the data collection, through a careful selection of the attitudinal questions included in the questionnaire.

## Supporting information

S1 File (DOCX)

S2 File (DOCX)

## References

[1] Adamowicz W, Louviere J, Williams M. Combining revealed and stated preference methods for valuing environmental amenities. J Environ Econ Manage. 1994; 26, 271–292. 10.1006/jeem.1994.1017.

[2] Adamowicz W, Swait J, Boxall P, Louviere J, Williams M. Perceptions versus objective measures of environmental quality in combined revealed and stated preference models of environmental valuation. 1997; J Environ Econ Manage. 32, 65–84. 10.1006/jeem.1996.0957

[3] Alberini A, Longo A, Tonin S, Trombetta F, Turvani M. The role of liability, regulation and economic incentives in brownfield remediation and redevelopment: evidence from surveys of developers. Reg Sci Urban Econ. 2005; 35, 327–351. 10.1016/j.regsciurbeco.2004.05.004.

[4] Longo A, Markandya A, Petrucci M. The internalization of externalities in the production of electricity: willingness to pay for the attributes of a policy for renewable energy. Ecol Econ. 2008; 67, 140–52. 10.1016/j.ecolecon.2007.12.006.

[5] Birol E, Smale M, Gyovai Á. Using a choice experiment to estimate farmers’ valuation of agrobiodiversity on Hungarian small farms. Environ Resour Econ. 2006; 34, 439–469. 10.1007/s10640-006-0009-9.

[6] Aguilar FX, Cai Z. Conjoint effect of environmental labeling, disclosure of forest of origin and price on consumer preferences for wood products in the US and UK. Ecol Econ. 2010; 70, 308–316. 10.1016/j.ecolecon.2010.09.002.

[7] McFadden D, Train K. Mixed MNL Models for discrete response. J Appl Econ. 2000; 15, 447–470. 10.1002/1099-1255(200009/10)15:5<447::AID-JAE570>3.0.CO;2-1.

[8] Hess S, Train K. Correlation and scale in mixed logit models. J Choice Model. 2017; 23, 1–8. 10.1016/j.jocm.2017.03.001.

[9] Ben-Akiva M, McFadden D, Train K, Walker J, Bhat C, Bierlaire M, et al. Hybrid choice models: Progress and challenges. Mark Lett. 2002; 13, 163–175. 10.1023/A:1020254301302.

[10] Vij A, Walker JL. How, when and why Integrated Choice and Latent Variable models are latently useful. Transport Res B-Meth. 2016; 90, 192–217. 10.1016/j.trb.2016.04.021.

[11] Agimass F, Lundhede T, Panduro TE, Jacobsen JB. The choice of forest site for recreation: A revealed preference analysis using spatial data. Ecosyst Serv. 2017; 31, 445–454. 10.1016/j.ecoser.2017.11.016.

[12] Bronnmann J., Asche F., 2017. Sustainable seafood from aquaculture and wild fisheries: Insights from a discrete choice experiment in Germany. Ecol Econ, 142, 113–119. 10.1016/j.ecolecon.2017.06.005.

[13] Ropars-Collet C, Leplat M, Goffe PL. Commercial Fisheries as an Asset for Recreational Demand on the Coast: Evidence from a Choice Experiment. Mar Resour Econ. 2017; 32, 391–409. 10.1086/693022.

[14] Paltriguera L, Ferrini S, Luisetti T, Turner RK. An analysis and valuation of post-designation management aimed at maximising recreational benefits in coastal marine protected areas. Ecol Econ. 2018; 148, 121–130. 10.1016/j.ecolecon.2018.02.011.

[15] Barrowclough MJ, Alwang J. Conservation agriculture in Ecuador’s highlands: a discrete choice experiment. Environ Dev Sustain. 2017; 1–25. 10.1007/s10668-017-0011-0.

[16] Brouwer R, Bliem M, Getzner M, Kerekes S, Milton S, Palarie T, et al. Valuation and transferability of the non-market benefits of river restoration in the Danube river basin using a choice experiment. Ecological Engineering, 2016; 87, pp.20–29. 10.1016/j.ecoleng.2015.11.018.

[17] Fujino M, Kuriyama K, Yoshida K. An evaluation of the natural environment ecosystem preservation policies in Japan. J Forest Econ. 2017; 29, 62–67. 10.1016/j.jfe.2017.08.003.

[18] Dechasa F, Senbeta F, Guta D. Economic value of wetlands services in the Central Rift Valley of Ethiopia. Environ Econ Policy Stud. 2021; 23, 29–53. 10.1007/s10018-020-00277-4

[19] Huynh E, Araña J E, Prior J. Evaluating residents’ preferences for remediation technologies: A choice experiment approach, Science of The Total Environment. 2018; 21, 1012–1022, doi: 10.1016/j.scitotenv.2017.10.125 29122350

[20] Seeteram NA, Engel V, Mozumder P. Implications of a valuation study for ecological and social indicators associated with Everglades restoration, Science of The Total Environment. 2018; 627, 792–801, doi: 10.1016/j.scitotenv.2018.01.152 29426204

[21] Alberini A, Bigano A, Ščasný M, Zvěřinová I. Preferences for energy efficiency vs. renewables: what is the willingness to pay to reduce CO2 emissions? Ecol Econ. 2018; 144, 171–185. 10.1016/j.ecolecon.2017.08.009.

[22] Waldman KB, Ortega DL, Richardson RB, Snapp SS. Estimating demand for perennial pigeon pea in Malawi using choice experiments. Ecol Econ. 2017; 131, 222–230. doi: 10.1016/j.ecolecon.2016.09.006 28050117 PMC5127491

[23] Wakamatsu M, Shin KJ, Wilson C, Managi S. Exploring a gap between Australia and Japan in the economic valuation of whale conservation. Ecol Econ. 2018; 146, 397–407. 10.1016/j.ecolecon.2017.12.002.

[24] Mariel P, Meyerhoff J. A More Flexible Model or Simply More Effort? On the Use of Correlated Random Parameters in Applied Choice Studies. Ecol Econ, 2018; 154, 419–429. 10.1016/j.ecolecon.2018.08.020

[25] Bjørnåvold A, Lizin S, Van Dael M, Arnold F, Van Passel S. Eliciting policymakers’ preferences for technologies to decarbonise transport: A discrete choice experiment, Environmental Innovation and Societal Transitions. 2020; 35, 21–34, 10.1016/j.eist.2019.12.002.

[26] Hess S, Beharry-Borg N. Accounting for latent attitudes in willingness-to-pay studies: The case of coastal water quality improvements in Tobago. Environ Resour Econ. 2012; 52, 109–131. 10.1007/s10640-011-9522-6.

[27] Dekker T, Hess S, Arentze T, Chorus C. Incorporating needs-satisfaction in a discrete choice model of leisure activities. J Transp Geogr. 2014; 38, 66–74. 10.1016/j.jtrangeo.2014.05.015.

[28] Bartczak A, Mariel P, Chilton S, Meyerhoff J. The impact of latent risk preferences on valuing preservation of threatened lynx populations in Poland, forthcoming in Aust J Agric Resour Econ. 2015; 60, 284–306. 10.1111/1467-8489.12123.

[29] Hoyos D, Mariel P, Hess S. Incorporating environmental attitudes in discrete choice models: An exploration of the utility of the awareness of consequences scale. Sci Total Environ. 2015; 505, 1100–1111. doi: 10.1016/j.scitotenv.2014.10.066 25461111

[30] Mariel P, Meyerhoff J, Hess S. Heterogeneous preferences toward landscape externalities of wind turbines—combining choices and attitudes in a hybrid model. Ren & Sust En Rev. 2015; 41, 647–657. 10.1016/j.rser.2014.08.074.

[31] Lundhede TH, Jacobsen JB, Hanley N, Strange N, Thorsen BJ. Incorporating outcome uncertainty and prior outcome beliefs in stated preferences. Land Econ. 2015; 91, 296–316. 10.3368/le.91.2.296.

[32] Mariel P, Meyerhoff J. Hybrid discrete choice models: Gained insights versus increasing effort. Sci Total Environ. 2016; 568, 433–443. doi: 10.1016/j.scitotenv.2016.06.019 27310534

[33] Taye FA, Vedel SE, Jacobsen JB. Accounting for environmental attitude to explain variations in willingness to pay for forest ecosystem services using the new environmental paradigm. J Env Econ & Policy. 2018; 7, 420–440. 10.1080/21606544.2018.1467346.

[34] Faccioli M, Czajkowski M, Glenk K, Martin-Ortega J. Environmental attitudes and place identity as determinants of preferences for ecosystem services. Ecological Economics. 2020; 174, 106600. 10.1016/j.ecolecon.2020.106600

[35] Owusu-Sekyere E, Abdulai A, Jordaan H. et al. Heterogeneous demand for ecologically sustainable products on ensuring environmental sustainability in South Africa. Environ Econ Policy Stud. 2020; 22, 39–64. 10.1007/s10018-019-00246-6

[36] Chorus C, Kroesen M. On the (im-)possibility of deriving transport policy implications from hybrid choice models. Transp Policy. 2014; 36, 217–222. 10.1016/j.tranpol.2014.09.001.

[37] Bahamonde-Birke FJ, Kunert U, Link H, Ortúzar J. About attitudes and perceptions: Finding the proper way to consider latent variables in discrete choice models. Transportation. 2017; 44(3), 475–493.

[38] Borriello A, Rose JM. Global versus localised attitudinal responses in discrete choice. Transportation. 2019; 48, 131–165. 10.1007/s11116-019-10045-3

[39] McFadden D. Conditional logit analysis of qualitative choice behaviour, in: Zarembka P. (Ed.), Frontiers in Econometrics. Academic Press. New York. 1974; 105–142.

[40] Train K. Discrete choice methods with simulation, second ed. Cambridge University Press, Cambridge, MA. 2009.

[41] Daly A, Hess S, Patruni B, Potoglou D, Rohr C. Using ordered attitudinal indicators in a latent variable choice model: A study of the impact of security on rail travel behaviour. Transportation. 2012; 39(2), 267–297. 10.1007/s11116-011-9351-z.

[42] Hess S, Rose J. Can scale and coefficient heterogeneity be separated in random coefficients models? Transp. 2012; 39, 1225–1239. https://doi.org/10.1007%2Fs11116-012-9394-9.

[43] Train K, Weeks M. Discrete choice models in preference space and willingness-to-pay space, in: Scarpa R., Alberini A. (Eds.), Applications of Simulation Methods in Environmental and Resource Economics. Springer, The Netherlands: Dordrecht. 2005; 1–16. 10.1007/1-4020-3684-1_1.

[44] Hess S, Stathopoulos A. Linking response quality to survey engagement: a combined random scale and latent variable approach. J Choice Model. 2013; 7, 1–12. 10.1016/j.jocm.2013.03.005.

[45] Bierlaire M. PythonBiogeme: a short introduction. Report TRANSP-OR 160706, Series on Biogeme. Transport and Mobility Laboratory, School of Architecture, Civil and Environmental Engineering, Ecole Polytechnique Fédérale de Lausanne, Switzerland. 2016.

[46] Rose JM, Bliemer MCJ. Ngene. http://www.choice-metrics.com/download.html. [Accessed September, 2017].

[47] Eurobarometer (EB), 2017: Special Eurobarometer 459/Wave EB 87.1: Climate Change. Brussels: European Commission. https://ec.europa.eu/clima/sites/clima/files/support/docs/report_2017_en.pdf

[48] Eurobarometer (EB), 2014: Special Eurobarometer 416/Wave EB 81.3: Attitudes Of European Citizens Towards The Environment. Brussels: European Commission.http://ec.europa.eu/commfrontoffice/publicopinion/archives/ebs/ebs_416_en.pdf

[49] Demel S, Longo A, Mariel P. Trading off visual disamenity for renewable energy: Willingness to pay for seaweed farming for energy production. Ecological Economics, 2020; 173, 106650. 10.1016/j.ecolecon.2020.106650

[50] Office for National Statistics: Census 2011 Table Links: Key Statistics at: http://www.nomisweb.co.uk/census/2011/key_statistics. [Accessed March, 2018].

[51] National Records of Scotland: Scotland’s Census 2011—National Records of Scotland Table KS102SC—Age structure http://www.scotlandscensus.gov.uk/ods-analyser/jsf/tableView/tableView.xhtml [Accessed March, 2018].

[52] Northern Ireland Statistics and Research Agency: Census 2011: Key Statistics for Northern Ireland at: https://www.nisra.gov.uk/sites/nisra.gov.uk/files/publications/2011-census-results-key-statistics-summary-report.pdf. [Accessed March, 2018].

[53] Sarrias M, Daziano R. Multinomial Logit Models with Continuous and Discrete Individual Heterogeneity in R: The gmnl Package. J Stat Softw. 2017; 79, 1–46. 10.18637/jss.v079.i02. doi: 10.18637/jss.v079.i02 30220889

[54] Bierlaire M. BIOGEME: a free package for the estimation of discrete choice models. Proceedings of the 3rd Swiss Transportation Research Conference. 2003.

[55] Hess S, Palma D. Apollo: A flexible, powerful and customisable freeware package for choice model estimation and application—ScienceDirect. Journal of Choice Modelling. 2019; 32, 100170.2019.100170. 10.1016/j.jocm

[56] Sheffield, U. of, n.d. Stata resources—Arne Risa Hole—Our people—Economics—The University of Sheffield [WWW Document]. URL https://www.sheffield.ac.uk/economics/people/hole/stata (accessed August 2019).

[57] Czajkowski M. Models for Discrete Choice Experiments [WWW Document]. URL http://czaj.org/research/estimation-packages/dce (accessed March 2020).

[58] Dunlap RE, Liere KDV. The ‘New Environmental Paradigm’. The Journal of Environmental Education. 2008; 40(1), 19–28. 10.3200/JOEE.40.1.19-28

