# Supplementary material for: Modelling welfare estimates in discrete choice experiments for seaweed-based renewable energy
Source: PLoS One. 2021 Nov 29;16(11):e0260352. doi: 10.1371/journal.pone.0260352 (PMC8629263; doi:10.1371/journal.pone.0260352)
Supplement: S1 File — (DOCX) [file pone.0260352.s001.docx]

**Supporting information**

S1 Table A1: Remainder of Model Estimates from Table 6 for England

|  | **Uncorrelated RPL** | | | **Correlated RPL** | | | | **Hybrid Choice Model** | | | | | | | | | |
| --- | --- | --- | --- | --- | --- | --- | --- | --- | --- | --- | --- | --- | --- | --- | --- | --- | --- |
|  | Coefficient | | St. Error | Coefficient | | | St. Error | Coefficient | | | | | St. Error | | | | |
| *Interaction effects* |  | |  |  | | |  |  | | | | | | | | | |
| 85,000 households powered ($\beta_{1}$) |  | |  |  | | |  |  | | | | | | | | | |
| Age | 0.012 | *** | (0.005) | 0.015 | *** | | (0.005) | 0.012 | | | | | ** | | (0.005) | | |
| Female | 0.087 |  | (0.121) | 0.080 |  | | (0.129) | 0.089 | | | | |  | | (0.121) | | |
| Cohabitating partnership | 0.032 |  | (0.136) | -0.186 |  | | (0.136) | 0.021 | | | | |  | | (0.135) | | |
| Number of children | -0.111 | * | (0.059) | -0.054 |  | | (0.062) | -0.118 | | | | | ** | | (0.059) | | |
| High education | 0.214 | * | (0.121) | -0.043 |  | | (0.134) | 0.190 | | | | |  | | (0.122) | | |
| Employed | -0.218 |  | (0.134) | -0.016 |  | | (0.149) | -0.223 | | | | | * | | (0.134) | | |
| Buys green energy | -0.001 |  | (0.155) | -0.117 |  | | (0.173) | -0.040 | | | | |  | | (0.163) | | |
| Political orientation | -0.114 | *** | (0.029) | -0.108 | *** | | (0.032) | -0.116 | | | | | *** | | (0.029) | | |
| High income | 0.543 | *** | (0.137) | 0.127 |  | | (0.159) | 0.540 | | | | | *** | | (0.138) | | |
| Latent variable |  |  |  |  |  | |  | -0.062 | | | | |  | | (0.082) | | |
| 130,000 households powered ($\beta_{2}$) |  | |  |  | | |  |  | | | | |  | | | | |
| Age | 0.022 | *** | (0.007) | 0.023 | *** | | (0.007) | 0.023 | | | | | *** | | | (0.007) | |
| Female | 0.262 |  | (0.167) | 0.186 |  | | (0.185) | 0.282 | | | | | * | | | (0.167) | |
| Cohabitating partnership | -0.275 |  | (0.189) | -0.201 |  | | (0.193) | -0.275 | | | | |  | | | (0.190) | |
| Number of children | 0.092 |  | (0.088) | 0.093 |  | | (0.090) | 0.072 | | | | |  | | | (0.089) | |
| High education | 0.273 |  | (0.174) | -0.069 |  | | (0.191) | 0.265 | | | | |  | | | (0.177) | |
| Employed | -0.293 |  | (0.195) | -0.256 |  | | (0.212) | -0.298 | | | | |  | | | (0.197) | |
| Buys green energy | 0.101 |  | (0.219) | -0.291 |  | | (0.248) | -0.012 | | | | |  | | | (0.233) | |
| Political orientation | -0.143 | *** | (0.041) | -0.138 | *** | | (0.045) | -0.153 | | | | | *** | | | (0.042) | |
| High income | 0.751 | *** | (0.205) | 0.307 |  | | (0.228) | 0.767 | | | | | *** | | | (0.210) | |
| Latent variable |  |  |  |  |  | |  | -0.170 | | | | |  | | | (0.116) | |
| 20% of coastline used ($\beta_{3}$) |  | |  |  | | |  |  | | | | | | | | | |
| Age | -0.009 | * | (0.005) | -0.006 |  | | (0.005) | -0.010 | | | | | ** | | | (0.005) | |
| Female | -0.108 |  | (0.116) | -0.070 |  | | (0.120) | -0.105 | | | | |  | | | (0.117) | |
| Cohabitating partnership | 0.125 |  | (0.132) | -0.246 | * | | (0.126) | 0.117 | | | | |  | | | (0.133) | |
| Number of children | -0.016 |  | (0.055) | 0.003 |  | | (0.058) | -0.009 | | | | |  | | | (0.057) | |
| High education | 0.030 |  | (0.115) | 0.062 |  | | (0.125) | 0.043 | | | | |  | | | (0.118) | |
| Employed | -0.082 |  | (0.135) | 0.142 |  | | (0.137) | -0.057 | | | | |  | | | (0.136) | |
| Buys green energy | -0.004 |  | (0.140) | 0.051 |  | | (0.162) | 0.055 | | | | |  | | | (0.145) | |
| Political orientation | 0.014 |  | (0.027) | 0.012 |  | | (0.030) | 0.022 | | | | |  | | | (0.027) | |
| High income | -0.141 |  | (0.138) | -0.069 |  | | (0.148) | -0.160 | | | | |  | | | (0.142) | |
| Latent variable |  |  |  |  |  | |  | 0.090 | | | | |  | | | (0.080) | |
| 30% of coastline used ($\beta_{4}$) |  | |  |  | | |  |  |  | | | |  | | |  | |
| Age | -0.006 |  | (0.006) | -0.004 |  | | (0.006) | -0.006 | | | | |  | | | (0.006) | |
| Female | -0.132 |  | (0.137) | -0.077 |  | | (0.148) | -0.128 | | | | |  | | | (0.137) | |
| Cohabitating partnership | -0.002 |  | (0.152) | -0.095 |  | | (0.155) | 0.020 | | | | |  | | | (0.152) | |
| Number of children | 0.072 |  | (0.067) | 0.052 |  | | (0.071) | 0.049 | | | | |  | | | (0.066) | |
| High education | -0.032 |  | (0.139) | 0.051 |  | | (0.153) | -0.061 | | | | |  | | | (0.142) | |
| Employed | -0.061 |  | (0.163) | -0.061 |  | | (0.168) | -0.054 | | | | |  | | | (0.166) | |
| Buys green energy | -0.142 |  | (0.182) | -0.139 |  | | (0.198) | -0.207 | | | | |  | | | (0.182) | |
| Political orientation | 0.008 |  | (0.034) | -0.006 |  | | (0.036) | 0.011 | | | | |  | | | (0.034) | |
| High income | -0.174 |  | (0.171) | -0.231 |  | | (0.181) | -0.190 | | | | |  | | | (0.173) | |
| Latent variable |  |  |  |  |  | |  | -0.083 | | | | |  | | | (0.085) | |
| Cost ($\beta_{5}$) |  | |  |  | | |  |  |  | | | |  | | |  | |
| Age | 0.030 | *** | (0.004) | 0.033 | *** | | (0.004) | 0.030 | | | | | *** | | | (0.004) | |
| Female | 0.225 | ** | (0.093) | 0.261 | *** | | (0.092) | 0.191 | | | | | * | | | (0.104) | |
| Cohabitating partnership | -0.036 |  | (0.097) | -0.133 |  | | (0.095) | -0.139 | | | | |  | | | (0.100) | |
| Number of children | -0.056 |  | (0.047) | -0.077 | * | | (0.042) | -0.014 | | | | |  | | | (0.057) | |
| High education | 0.035 |  | (0.093) | 0.173 | * | | (0.094) | 0.120 | | | | |  | | | (0.115) | |
| Employed | 0.069 |  | (0.110) | -0.116 |  | | (0.097) | 0.035 | | | | |  | | | (0.112) | |
| Buys green energy | -0.779 | *** | (0.155) | -0.138 |  | | (0.130) | -0.427 | | | | | *** | | | (0.137) | |
| Political orientation | -0.064 | *** | (0.020) | -0.076 | *** | | (0.022) | -0.049 | | | | | ** | | | (0.022) | |
| High income | -0.102 |  | (0.111) | -0.026 |  | | (0.108) | -0.186 | | | | |  | | | (0.146) | |
| Latent variable |  |  |  |  |  | |  | 0.461 | | | | | *** | | | (0.078) | |
| Letter with contribution perk ($\beta_{6}$) |  | |  |  | | |  |  |  | | | |  | | |  | |
| Age | -0.008 | * | (0.004) | -0.008 |  | | (0.005) | -0.008 | | | | | * | | | (0.004) | |
| Female | -0.003 |  | (0.111) | -0.010 |  | | (0.119) | -0.010 | | | | |  | | | (0.111) | |
| Cohabitating partnership | 0.050 |  | (0.122) | -0.056 |  | | (0.125) | 0.032 | | | | |  | | | (0.121) | |
| Number of children | -0.013 |  | (0.056) | 0.024 |  | | (0.058) | -0.009 | | | | |  | | | (0.056) | |
| High education | -0.059 |  | (0.118) | 0.045 |  | | (0.123) | -0.056 | | | | |  | | | (0.117) | |
| Employed | 0.232 | * | (0.128) | 0.052 |  | | (0.135) | 0.214 | | | | | * | | | (0.128) | |
| Buys green energy | -0.136 |  | (0.156) | 0.019 |  | | (0.160) | -0.145 | | | | |  | | | (0.158) | |
| Political orientation | -0.003 |  | (0.028) | -0.015 |  | | (0.029) | -0.003 | | | | |  | | | (0.028) | |
| High income | 0.033 |  | (0.127) | 0.008 |  | | (0.146) | 0.028 | | | | |  | | | (0.127) | |
| Latent variable |  |  |  |  |  | |  | -0.001 | | | | |  | | | (0.070) | |
| Facebook profile picture perk ($\beta_{7}$) |  | |  |  | | |  |  | | | | |  | | |  | |
| Age | -0.004 |  | (0.005) | -0.007 |  | | (0.005) | -0.004 | | | | |  | | | (0.005) | |
| Female | -0.006 |  | (0.124) | -0.052 |  | | (0.133) | -0.004 | | | | |  | | | (0.124) | |
| Cohabitating partnership | -0.115 |  | (0.135) | 0.019 |  | | (0.141) | -0.135 | | | | |  | | | | (0.135) |
| Number of children | 0.042 |  | (0.058) | 0.077 |  | | (0.065) | 0.058 | | | | |  | | | | (0.059) |
| High education | -0.012 |  | (0.130) | -0.082 |  | | (0.139) | -0.004 | | | | |  | | | | (0.130) |
| Employed | 0.263 | * | (0.144) | -0.031 |  | | (0.152) | 0.257 | | | | | * | | | | (0.144) |
| Buys green energy | -0.022 |  | (0.169) | 0.013 |  | | (0.180) | -0.020 | | | | |  | | | | (0.171) |
| Political orientation | 0.040 |  | (0.032) | 0.036 |  | | (0.032) | 0.040 | | | | |  | | | | (0.032) |
| High income | 0.223 |  | (0.140) | -0.014 |  | | (0.163) | 0.222 | | | | |  | | | | (0.140) |
| Latent variable |  |  |  |  |  | |  | 0.025 | | | | |  | | | | (0.082) |
|  |  | |  |  | | |  |  | | | | |  | | | | |
| *Elements of* $\Gamma$ |  | |  |  | | |  | *Structural equation for latent variable model:* | | | | | | | | | |
| 85,000 households, 85,000 households | | |  | 1.055 | *** | (0.084) | | Age | | | 0.001 | |  | | | | (0.004) |
| 85,000 households, 130,000 households | | |  | 1.768 | *** | (0.121) | | Female | | | 0.087 | |  | | | | (0.089) |
| 85,000 households, 20% of coastline | | |  | 0.083 |  | (0.102) | | Cohabit. part. | | | 0.022 | |  | | | | (0.091) |
| 85,000 households, 30% of coastline | | |  | -0.092 |  | (0.128) | | No. of children | | | -0.090 | | ** | | | | (0.045) |
| 85,000 households, cost | | |  | 0.104 | * | (0.056) | | High educ. | | | -0.259 | | *** | | | | (0.095) |
| 85,000 households, letter with contribution | | |  | 0.377 | *** | (0.094) | | Employed | | | -0.083 | |  | | | | (0.101) |
| 85,000 households, Facebook profile picture | | |  | 0.517 | *** | (0.109) | | Buys green en. | | | -0.646 | | *** | | | | (0.124) |
| 130,000 households, 130,000 households | | |  | 0.816 | *** | (0.133) | | Pol. orientation | | | -0.028 | |  | | | | (0.022) |
| 130,000 households, 20% of coastline | | |  | -0.634 | *** | (0.147) | | High income | | | 0.075 | |  | | | | (0.120) |
| 130,000 households, 30% of coastline | | |  | -0.643 | *** | (0.186) | |  | | |  | |  | | | | |
| 130,000 households, cost | | |  | -0.234 | *** | (0.064) | | *Measurement equation parameters:* | | | | | | | | | |
| 130,000 households, letter with contribution | | |  | -0.284 | * | (0.152) | | $\zeta_{1}$ | | | | 2.074 | | *** | | | (0.216) |
| 130,000 households, Facebook profile picture | | |  | -0.658 | *** | (0.142) | | $\zeta_{2}$ | | | | 1.068 | | *** | | | (0.143) |
| 20% of coastline, 20% of coastline | | |  | 0.781 | *** | (0.120) | | $\zeta_{3}$ | | | | 1.142 | | *** | | | (0.144) |
| 20% of coastline, 30% of coastline | | |  | 1.151 | *** | (0.160) | | $\zeta_{4}$ | | | | 1.936 | | *** | | | (0.211) |
| 20% of coastline, cost | | |  | 0.173 | * | (0.100) | | $\zeta_{5}$ | | | | 1.664 | | *** | | | (0.173) |
| 20% of coastline, letter with contribution | | |  | 0.342 | ** | (0.161) | | $\zeta_{6}$ | | | | 1.187 | | *** | | | (0.125) |
| 20% of coastline, Facebook profile picture | | |  | 0.001 |  | (0.166) | | $\zeta_{7}$ | | | | 0.543 | | *** | | | (0.093) |
| 30% of coastline, 30% of coastline | | |  | -0.554 | *** | (0.170) | |  | | | | | | | | | |
| 30% of coastline, cost | | |  | 0.326 | *** | (0.102) | |  | | | | | | | | | |
| 30% of coastline, letter with contribution | | |  | 0.301 |  | (0.188) | | $\tau_{1}$ | | | | -2.734 | *** | | | | (0.474) |
| 30% of coastline, Facebook profile picture | | |  | 0.032 |  | (0.233) | | $\delta_{11}$ | | | | 3.620 | *** | | | | (0.251) |
| cost, cost | | |  | 1.035 | *** | (0.045) | | $\delta_{21}$ | | | | 3.982 | *** | | | | (0.369) |
| cost, letter with contribution | | |  | -0.157 |  | (0.105) | | $\tau_{3}$ | | | | -1.837 | *** | | | | (0.269) |
| cost, Facebook profile picture | | |  | -0.133 |  | (0.117) | | $\delta_{13}$ | | | | 2.690 | *** | | | | (0.149) |
| letter with contribution, letter with contribution | | | | -0.466 | ** | (0.205) | | $\delta_{23}$ | | | | 2.986 | *** | | | | (0.237) |
| letter with contribution, Facebook profile picture | | | | -0.394 |  | (0.253) | | $\tau_{4}$ | | | | -0.823 | ** | | | | (0.387) |
| Facebook profile picture, Facebook profile picture | | | | -0.190 |  | (0.237) | | $\delta_{14}$ | | | | 4.391 | *** | | | | (0.293) |
|  | | | |  |  |  | | $\delta_{24}$ | | 2.902 | | | *** | | | | (0.516) |
|  | | | |  |  |  | | $\tau_{5}$ | | -2.580 | | | *** | | | | (0.354) |
|  | | | |  |  |  | | $\delta_{15}$ | | 3.770 | | | *** | | | | (0.230) |
|  | | | |  |  |  | | $\delta_{25}$ | | 2.899 | | | *** | | | | (0.242) |
|  | | | |  |  |  | | $\tau_{6}$ | | -0.816 | | | *** | | | | (0.248) |
|  | | | |  |  |  | | $\delta_{16}$ | | 3.265 | | | *** | | | | (0.164) |
|  | | | |  |  |  | | $\delta_{26}$ | | 2.749 | | | *** | | | | (0.403) |
|  | | | |  |  |  | | $\tau_{7}$ | | 0.189 | | |  | | | | (0.135) |
|  | | | |  |  |  | | $\delta_{17}$ | | 2.664 | | | *** | | | | (0.157) |
|  | | | |  |  |  | | $\delta_{27}$ | | 2.049 | | | *** | | | | (0.385) |
|  | | | |  |  |  | |  | |  | | |  | | | |  |

Note: ***, **, * denote significance at the 1%, 5%, and 10% level, respectively.

S1 Table A2: Remainder of Model Estimates from Table 7 for Scotland

|  | **Uncorrelated RPL** | | | **Correlated RPL** | | | | **Hybrid Choice Model** | | | | | | | |
| --- | --- | --- | --- | --- | --- | --- | --- | --- | --- | --- | --- | --- | --- | --- | --- |
|  | Coefficient | | Standard Error | Coefficient | | | Standard Error | Coefficient | | Standard Error | | | | | |
| *Interaction effects* |  | |  |  | | |  |  | | | | | | | |
| 85,000 households powered ($\beta_{1}$) |  | |  |  | | |  |  | | | | | | | |
| Age | 0.012 | * | (0.006) | 0.012 | * | | (0.007) | 0.012 | | * | | (0.006) | | | |
| Female | -0.327 | ** | (0.154) | -0.206 |  | | (0.174) | -0.339 | | ** | | (0.158) | | | |
| Cohabitating partnership | -0.009 |  | (0.186) | -0.071 |  | | (0.186) | -0.037 | |  | | (0.187) | | | |
| Number of children | -0.021 |  | (0.084) | -0.047 |  | | (0.084) | -0.016 | |  | | (0.087) | | | |
| High education | -0.139 |  | (0.164) | 0.114 |  | | (0.180) | -0.137 | |  | | (0.164) | | | |
| Employed | -0.280 |  | (0.187) | 0.155 |  | | (0.200) | -0.273 | |  | | (0.188) | | | |
| Buys green energy | 0.085 |  | (0.204) | -0.106 |  | | (0.241) | -0.002 | |  | | (0.216) | | | |
| Political orientation | -0.122 | ** | (0.050) | -0.128 | ** | | (0.051) | -0.112 | | ** | | (0.053) | | | |
| High income | -0.115 |  | (0.187) | -0.214 |  | | (0.216) | -0.157 | |  | | (0.190) | | | |
| Latent variable |  |  |  |  |  | |  | -0.110 | |  | | (0.111) | | | |
| 130,000 households powered ($\beta_{2}$) |  | |  |  | | |  |  | | | | | | | |
| Age | 0.014 |  | (0.009) | 0.014 |  | | (0.010) | 0.012 | |  | | | | (0.010) | |
| Female | -0.077 |  | (0.239) | -0.025 |  | | (0.245) | -0.090 | |  | | | | (0.240) | |
| Cohabitating partnership | -0.025 |  | (0.266) | -0.056 |  | | (0.261) | -0.034 | |  | | | | (0.269) | |
| Number of children | -0.141 |  | (0.111) | -0.157 |  | | (0.120) | -0.142 | |  | | | | (0.115) | |
| High education | -0.154 |  | (0.243) | 0.463 | * | | (0.257) | -0.120 | |  | | | | (0.251) | |
| Employed | -0.221 |  | (0.289) | 0.413 |  | | (0.280) | -0.224 | |  | | | | (0.296) | |
| Buys green energy | 0.050 |  | (0.331) | -0.107 |  | | (0.342) | -0.162 | |  | | | | (0.392) | |
| Political orientation | -0.175 | ** | (0.073) | -0.175 | ** | | (0.073) | -0.150 | | ** | | | | (0.073) | |
| High income | 0.163 |  | (0.272) | -0.505 | * | | (0.307) | 0.072 | |  | | | | (0.296) | |
| Latent variable |  |  |  |  |  | |  | -0.275 | |  | | | | (0.181) | |
| 20% of coastline used ($\beta_{3}$) |  | |  |  | | |  |  | | | | | | | |
| Age | -0.006 |  | (0.006) | -0.006 |  | | (0.006) | -0.005 | |  | | | | (0.006) | |
| Female | 0.022 |  | (0.156) | 0.045 |  | | (0.171) | 0.028 | |  | | | | (0.159) | |
| Cohabitating partnership | -0.221 |  | (0.190) | 0.102 |  | | (0.178) | -0.183 | |  | | | | (0.193) | |
| Number of children | 0.052 |  | (0.081) | -0.005 |  | | (0.081) | 0.048 | |  | | | | (0.082) | |
| High education | -0.103 |  | (0.167) | -0.239 |  | | (0.175) | -0.145 | |  | | | | (0.169) | |
| Employed | 0.024 |  | (0.181) | -0.011 |  | | (0.197) | 0.054 | |  | | | | (0.186) | |
| Buys green energy | -0.068 |  | (0.211) | 0.267 |  | | (0.234) | -0.051 | |  | | | | (0.233) | |
| Political orientation | 0.055 |  | (0.044) | 0.028 |  | | (0.050) | 0.048 | |  | | | (0.046) | | |
| High income | -0.187 |  | (0.186) | 0.180 |  | | (0.207) | -0.204 | |  | | | | (0.194) | |
| Latent variable |  |  |  |  |  | |  | 0.021 | |  | | | | (0.097) | |
| 30% of coastline used ($\beta_{4}$) |  | |  |  | | |  |  | |  | | | |  | |
| Age | -0.009 |  | (0.009) | -0.007 |  | | (0.009) | -0.009 | |  | | | | (0.009) | |
| Female | 0.118 |  | (0.210) | 0.094 |  | | (0.229) | 0.098 | |  | | | | (0.211) | |
| Cohabitating partnership | 0.088 |  | (0.256) | 0.068 |  | | (0.243) | 0.105 | |  | | | | (0.258) | |
| Number of children | 0.065 |  | (0.102) | 0.066 |  | | (0.112) | 0.062 | |  | | | | (0.104) | |
| High education | -0.303 |  | (0.219) | -0.262 |  | | (0.238) | -0.283 | |  | | | | (0.226) | |
| Employed | 0.238 |  | (0.245) | 0.114 |  | | (0.269) | 0.283 | |  | | | | (0.253) | |
| Buys green energy | -0.092 |  | (0.278) | -0.052 |  | | (0.319) | -0.033 | |  | | | | (0.296) | |
| Political orientation | -0.080 |  | (0.065) | -0.087 |  | | (0.067) | -0.080 | |  | | | | (0.069) | |
| High income | -0.176 |  | (0.241) | 0.082 |  | | (0.279) | -0.165 | |  | | | | (0.244) | |
| Latent variable |  |  |  |  |  | |  | 0.055 | |  | | | | (0.126) | |
| Cost ($\beta_{5}$) |  | |  |  | | |  |  | |  | | | |  | |
| Age | 0.024 | *** | (0.006) | 0.022 | *** | | (0.005) | 0.023 | | ** | | | | (0.010) | |
| Female | -0.315 | ** | (0.136) | -0.179 |  | | (0.141) | -0.281 | |  | | | | (0.264) | |
| Cohabitating partnership | -0.310 | ** | (0.155) | 0.038 |  | | (0.147) | -0.281 | | * | | | | (0.156) | |
| Number of children | -0.019 |  | (0.070) | -0.096 |  | | (0.063) | -0.016 | |  | | | | (0.099) | |
| High education | -0.156 |  | (0.140) | -0.016 |  | | (0.129) | -0.103 | |  | | | | (0.173) | |
| Employed | -0.249 | * | (0.144) | 0.011 |  | | (0.148) | -0.339 | | * | | | | (0.189) | |
| Buys green energy | -0.200 |  | (0.170) | -0.115 |  | | (0.196) | 0.034 | |  | | | | (0.251) | |
| Political orientation | -0.019 |  | (0.044) | -0.005 |  | | (0.045) | -0.058 | |  | | | | (0.036) | |
| High income | -0.254 |  | (0.197) | 0.008 |  | | (0.179) | -0.251 | |  | | | | (0.296) | |
| Latent variable |  |  |  |  |  | |  | 0.282 | | * | | | | (0.152) | |
| Letter with contribution perk ($\beta_{6}$) |  | |  |  | | |  |  | |  | | | |  | |
| Age | -0.008 |  | (0.006) | -0.009 |  | | (0.007) | -0.008 | |  | | | | (0.006) | |
| Female | 0.128 |  | (0.157) | 0.190 |  | | (0.173) | 0.124 | |  | | | | (0.158) | |
| Cohabitating partnership | 0.167 |  | (0.187) | 0.010 |  | | (0.181) | 0.170 | |  | | | | (0.190) | |
| Number of children | -0.100 |  | (0.088) | -0.060 |  | | (0.088) | -0.106 | |  | | | | (0.091) | |
| High education | 0.222 |  | (0.166) | -0.036 |  | | (0.181) | 0.222 | |  | | | | (0.170) | |
| Employed | -0.140 |  | (0.178) | 0.165 |  | | (0.199) | -0.148 | |  | | | | (0.181) | |
| Buys green energy | 0.010 |  | (0.246) | -0.142 |  | | (0.242) | -0.051 | |  | | | | (0.265) | |
| Political orientation | -0.016 |  | (0.053) | 0.000 |  | | (0.053) | -0.009 | |  | | | | (0.055) | |
| High income | -0.073 |  | (0.190) | -0.044 |  | | (0.212) | -0.081 | |  | | | | (0.192) | |
| Latent variable |  |  |  |  |  | |  | -0.068 | |  | | | | (0.105) | |
| Facebook profile picture perk ($\beta_{7}$) |  | |  |  | | |  |  | |  | | | |  | |
| Age | -0.009 |  | (0.007) | -0.013 |  | | 0.008 | -0.011 | |  | | | | (0.007) | |
| Female | 0.394 | ** | (0.193) | 0.462 | ** | | 0.204 | 0.370 | | * | | | (0.196) | | |
| Cohabitating partnership | 0.163 |  | (0.224) | 0.164 |  | | 0.217 | 0.157 | |  | | | (0.227) | | |
| Number of children | -0.012 |  | (0.104) | 0.075 |  | | 0.104 | -0.021 | |  | | | (0.110) | | |
| High education | 0.099 |  | (0.205) | 0.093 |  | | 0.214 | 0.091 | |  | | | (0.203) | | |
| Employed | 0.025 |  | (0.221) | -0.029 |  | | 0.236 | 0.055 | |  | | | (0.224) | | |
| Buys green energy | 0.069 |  | (0.264) | 0.166 |  | | 0.284 | -0.038 | |  | | | (0.280) | | |
| Political orientation | -0.008 |  | (0.063) | -0.032 |  | | 0.063 | 0.005 | |  | | | (0.066) | | |
| High income | -0.076 |  | (0.229) | -0.061 |  | | 0.251 | -0.117 | |  | | | (0.231) | | |
| Latent variable |  |  |  |  |  | |  | -0.129 | |  | | | (0.119) | | |
|  |  | |  |  | | |  |  | |  | | | | | |
| *Elements of* $\Gamma$ |  | |  |  | | |  | *Structural equation for latent variable model:* | | | | | | | |
| 85,000 households, 85,000 households | | |  | 0.756 | | *** | 0.116 | Age | -0.003 | |  | | | | (0.005) |
| 85,000 households, 130,000 households | | |  | 1.506 | | *** | 0.209 | Female | -0.170 | |  | | | | (0.130) |
| 85,000 households, 20% of coastline | | |  | -0.264 | |  | 0.164 | Cohabit. part. | -0.012 | |  | | | | (0.130) |
| 85,000 households, 30% of coastline | | |  | -0.181 | |  | 0.320 | No. of children | -0.046 | |  | | | | (0.061) |
| 85,000 households, cost | | |  | -0.337 | | * | 0.178 | High educ. | -0.006 | |  | | | | (0.136) |
| 85,000 households, letter with contribution | | |  | 0.057 | |  | 0.224 | Employed | 0.109 | |  | | | | (0.153) |
| 85,000 households, Facebook profile picture | | |  | 0.474 | |  | 0.296 | Buys green en. | -0.814 | | *** | | | | (0.219) |
| 130,000 households, 130,000 households | | |  | 0.691 | | *** | 0.256 | Pol. orientation | 0.102 | | *** | | | | (0.037) |
| 130,000 households, 20% of coastline | | |  | -0.111 | |  | 0.347 | High income | -0.151 | |  | | | | (0.189) |
| 130,000 households, 30% of coastline | | |  | -0.635 | |  | 0.589 |  | | |  | | | | |
| 130,000 households, cost | | |  | 0.354 | |  | 0.393 | *Measurement equation parameters:* | | | | | | | |
| 130,000 households, letter with contribution | | |  | -0.433 | |  | 0.355 | $\zeta_{1}$ | 1.892 | | *** | | | | (0.331) |
| 130,000 households, Facebook profile picture | | |  | -0.835 | | *** | 0.289 | $\zeta_{2}$ | 1.104 | | *** | | | | (0.240) |
| 20% of coastline, 20% of coastline | | |  | 0.824 | | *** | 0.154 | $\zeta_{3}$ | 1.056 | | *** | | | | (0.222) |
| 20% of coastline, 30% of coastline | | |  | 1.401 | | *** | 0.305 | $\zeta_{4}$ | 2.348 | | *** | | | | (0.411) |
| 20% of coastline, cost | | |  | 0.209 | |  | 0.130 | $\zeta_{5}$ | 1.737 | | *** | | | | (0.257) |
| 20% of coastline, letter with contribution | | |  | 0.383 | |  | 0.241 | $\zeta_{6}$ | 1.611 | | *** | | | | (0.223) |
| 20% of coastline, Facebook profile picture | | |  | 0.409 | |  | 0.440 | $\zeta_{7}$ | 0.731 | | *** | | | | (0.130) |
| 30% of coastline, 30% of coastline | | |  | 0.441 | |  | 0.375 |  | |  | | | | | |
| 30% of coastline, cost | | |  | -0.014 | |  | 0.189 |  | | | | | | | |
| 30% of coastline, letter with contribution | | |  | -0.584 | | ** | 0.295 | $\tau_{1}$ | -1.457 | | ** | | | | (0.597) |
| 30% of coastline, Facebook profile picture | | |  | -0.438 | |  | 0.395 | $\delta_{11}$ | 3.543 | | *** | | | | (0.373) |
| cost, cost | | |  | 0.984 | | *** | 0.163 | $\delta_{21}$ | 3.789 | | *** | | | | (0.504) |
| cost, letter with contribution | | |  | -0.021 | |  | 0.204 | $\tau_{2}$ | -0.524 | |  | | | | (0.345) |
| cost, Facebook profile picture | | |  | -0.006 | |  | 0.239 | $\delta_{12}$ | 2.796 | | *** | | | | (0.235) |
| letter with contribution, letter with contribution | | |  | -0.020 | |  | 0.361 | $\delta_{22}$ | 2.747 | | *** | | | | (0.400) |
| letter with contribution, Facebook profile picture | | |  | -0.050 | |  | 0.440 | $\tau_{3}$ | -1.195 | | *** | | | | (0.333) |
| Facebook profile picture, Facebook profile picture | | |  | -0.118 | |  | 0.290 | $\delta_{13}$ | 2.890 | | *** | | | | (0.230) |
|  | | |  |  | |  |  | $\delta_{23}$ | 3.108 | | *** | | | | (0.396) |
|  | | |  |  | |  |  | $\tau_{4}$ | 0.283 | |  | | | | (0.712) |
|  | | |  |  | |  |  | $\delta_{14}$ | 4.769 | | *** | | | | (0.602) |
|  | | |  |  | |  |  | $\delta_{24}$ | 3.166 | | *** | | | | (0.783) |
|  | | |  |  | |  |  | $\tau_{5}$ | -1.662 | | *** | | | | (0.518) |
|  | | |  |  | |  |  | $\delta_{15}$ | 3.921 | | *** | | | | (0.344) |
|  | | |  |  | |  |  | $\delta_{25}$ | 2.698 | | *** | | | | (0.328) |
|  | | |  |  | |  |  | $\tau_{6}$ | -0.141 | |  | | | | (0.496) |
|  | | |  |  | |  |  | $\delta_{16}$ | 3.814 | | *** | | | | (0.311) |
|  | | |  |  | |  |  | $\delta_{26}$ | 3.869 | | *** | | | | (1.000) |
|  | | |  |  | |  |  | $\tau_{7}$ | 0.431 | | * | | | | (0.242) |
|  | | |  |  | |  |  | $\delta_{17}$ | 2.952 | | *** | | | | (0.250) |
|  | | |  |  | |  |  | $\delta_{27}$ | 3.054 | | *** | | | | (0.977) |
|  | | |  |  | |  |  |  |  | |  | | | |  |

Note: ***, **, * denote significance at the 1%, 5%, and 10% level, respectively.

S1 Table A3: Remainder of Model Estimates from Table 8 for Northern Ireland

|  | **Uncorrelated RPL** | | | **Correlated RPL** | | | | **Hybrid Choice Model** | | | | | | | |
| --- | --- | --- | --- | --- | --- | --- | --- | --- | --- | --- | --- | --- | --- | --- | --- |
|  | Coefficient | | Standard Error | Coefficient | | | Standard Error | Coefficient | | Standard Error | | | | | |
| *Interaction effects* |  | |  |  | | |  |  | | | | | | | |
| 85,000 households powered ($\beta_{1}$) |  | |  |  | | |  |  | | | | | | | |
| Age | -0.001 |  | (0.007) | -0.005 |  | | (0.008) | -0.001 | |  | | (0.007) | | | |
| Female | 0.139 |  | (0.187) | -0.019 |  | | (0.212) | 0.112 | |  | | (0.184) | | | |
| Cohabitating partnership | -0.200 |  | (0.206) | -0.140 |  | | (0.224) | -0.206 | |  | | (0.202) | | | |
| Number of children | 0.073 |  | (0.082) | 0.054 |  | | (0.091) | 0.069 | |  | | (0.081) | | | |
| High education | 0.087 |  | (0.194) | 0.159 |  | | (0.216) | 0.073 | |  | | (0.195) | | | |
| Employed | 0.348 |  | (0.215) | 0.143 |  | | (0.234) | 0.303 | |  | | (0.205) | | | |
| Buys green energy | 0.189 |  | (0.238) | 0.326 |  | | (0.297) | 0.112 | |  | | (0.268) | | | |
| Political orientation | -0.004 |  | (0.040) | -0.004 |  | | (0.052) | -0.001 | |  | | (0.039) | | | |
| High income | 0.082 |  | (0.216) | -0.266 |  | | (0.275) | 0.111 | |  | | (0.217) | | | |
| Latent variable |  |  |  |  |  | |  | -0.130 | |  | | (0.140) | | | |
| 130,000 households powered ($\beta_{2}$) |  | |  |  | | |  |  | |  | | | | | |
| Age | 0.013 |  | (0.010) | 0.014 |  | | (0.011) | 0.015 | |  | | | | (0.010) | |
| Female | 0.163 |  | (0.274) | 0.034 |  | | (0.297) | 0.093 | |  | | | | (0.271) | |
| Cohabitating partnership | -0.015 |  | (0.296) | 0.209 |  | | (0.315) | -0.038 | |  | | | | (0.291) | |
| Number of children | 0.054 |  | (0.114) | 0.033 |  | | (0.129) | 0.028 | |  | | | | (0.111) | |
| High education | 0.199 |  | (0.275) | 0.423 |  | | (0.305) | 0.104 | |  | | | | (0.274) | |
| Employed | 0.534 | * | (0.304) | -0.487 |  | | (0.330) | 0.499 | | * | | | | (0.295) | |
| Buys green energy | -0.070 |  | (0.360) | 0.280 |  | | (0.414) | -0.274 | |  | | | | (0.393) | |
| Political orientation | -0.052 |  | (0.058) | -0.040 |  | | (0.074) | -0.056 | |  | | | | (0.056) | |
| High income | -0.219 |  | (0.331) | -0.374 |  | | (0.386) | -0.115 | |  | | | | (0.331) | |
| Latent variable |  |  |  |  |  | |  | -0.335 | |  | | | | (0.206) | |
| 20% of coastline used ($\beta_{3}$) |  | |  |  | | |  |  | | | | | | | |
| Age | -0.003 |  | (0.007) | -0.009 |  | | (0.007) | -0.002 | |  | | | | (0.007) | |
| Female | 0.090 |  | (0.177) | -0.028 |  | | (0.187) | 0.072 | |  | | | | (0.173) | |
| Cohabitating partnership | -0.293 |  | (0.194) | -0.005 |  | | (0.197) | -0.308 | |  | | | | (0.189) | |
| Number of children | -0.006 |  | (0.079) | -0.042 |  | | (0.081) | -0.013 | |  | | | | (0.078) | |
| High education | 0.184 |  | (0.180) | -0.297 |  | | (0.191) | 0.180 | |  | | | | (0.177) | |
| Employed | 0.182 |  | (0.213) | 0.463 | ** | | (0.207) | 0.169 | |  | | | | (0.209) | |
| Buys green energy | 0.277 |  | (0.202) | -0.115 |  | | (0.259) | 0.194 | |  | | | | (0.206) | |
| Political orientation | -0.039 |  | (0.044) | -0.066 |  | | (0.046) | -0.040 | |  | | | (0.044) | | |
| High income | -0.238 |  | (0.199) | 0.233 |  | | (0.240) | -0.199 | |  | | | | (0.191) | |
| Latent variable |  |  |  |  |  | |  | -0.071 | |  | | | | (0.110) | |
| 30% of coastline used ($\beta_{4}$) |  | |  |  | | |  |  | |  | | | |  | |
| Age | -0.011 |  | (0.007) | -0.017 | ** | | (0.008) | -0.011 | |  | | | | (0.007) | |
| Female | 0.078 |  | (0.193) | 0.054 |  | | (0.215) | 0.056 | |  | | | | (0.193) | |
| Cohabitating partnership | -0.277 |  | (0.197) | 0.188 |  | | (0.230) | -0.260 | |  | | | | (0.193) | |
| Number of children | 0.098 |  | (0.097) | 0.085 |  | | (0.091) | 0.092 | |  | | | | (0.096) | |
| High education | -0.081 |  | (0.181) | -0.112 |  | | (0.221) | -0.066 | |  | | | | (0.174) | |
| Employed | -0.055 |  | (0.202) | 0.426 | * | | (0.240) | -0.080 | |  | | | | (0.201) | |
| Buys green energy | 0.395 | * | (0.240) | 0.187 |  | | (0.302) | 0.392 | |  | | | | (0.248) | |
| Political orientation | -0.015 |  | (0.047) | -0.042 |  | | (0.053) | -0.023 | |  | | | | (0.048) | |
| High income | -0.407 | * | (0.229) | -0.043 |  | | (0.276) | -0.419 | | * | | | | (0.225) | |
| Latent variable |  |  |  |  |  | |  | 0.011 | |  | | | | (0.106) | |
| Cost ($\beta_{5}$) |  | |  |  | | |  |  | |  | | | |  | |
| Age | 0.020 | *** | (0.006) | 0.017 | *** | | (0.005) | 0.021 | | *** | | | | (0.006) | |
| Female | 0.318 | ** | (0.142) | 0.252 | * | | (0.144) | 0.339 | | ** | | | | (0.133) | |
| Cohabitating partnership | 0.043 |  | (0.154) | -0.168 |  | | (0.146) | 0.054 | |  | | | | (0.189) | |
| Number of children | -0.008 |  | (0.053) | 0.013 |  | | (0.062) | 0.052 | |  | | | | (0.063) | |
| High education | -0.225 | * | (0.137) | 0.048 |  | | (0.140) | -0.088 | |  | | | | (0.151) | |
| Employed | 0.071 |  | (0.163) | -0.026 |  | | (0.149) | 0.152 | |  | | | | (0.163) | |
| Buys green energy | -0.349 | * | (0.194) | 0.327 | * | | (0.180) | -0.109 | |  | | | | (0.183) | |
| Political orientation | 0.020 |  | (0.031) | 0.002 |  | | (0.033) | 0.015 | |  | | | | (0.028) | |
| High income | -0.322 | ** | (0.152) | 0.412 | ** | | (0.161) | -0.432 | | *** | | | | (0.164) | |
| Latent variable |  |  |  |  |  | |  | 0.285 | | ** | | | | (0.120) | |
| Letter with contribution perk ($\beta_{6}$) |  | |  |  | | |  |  | |  | | | |  | |
| Age | -0.008 |  | (0.008) | -0.007 |  | | (0.008) | -0.008 | |  | | | | (0.008) | |
| Female | -0.027 |  | (0.184) | -0.108 |  | | (0.208) | -0.081 | |  | | | | (0.175) | |
| Cohabitating partnership | 0.242 |  | (0.209) | -0.004 |  | | (0.221) | 0.211 | |  | | | | (0.200) | |
| Number of children | -0.150 | * | (0.084) | -0.111 |  | | (0.090) | -0.173 | | ** | | | | (0.081) | |
| High education | -0.408 | ** | (0.197) | -0.013 |  | | (0.213) | -0.462 | | ** | | | | (0.196) | |
| Employed | 0.136 |  | (0.212) | -0.142 |  | | (0.230) | 0.099 | |  | | | | (0.204) | |
| Buys green energy | -0.066 |  | (0.217) | 0.020 |  | | (0.295) | -0.295 | |  | | | | (0.231) | |
| Political orientation | 0.018 |  | (0.047) | 0.001 |  | | (0.051) | 0.022 | |  | | | | (0.047) | |
| High income | -0.268 |  | (0.217) | 0.668 | ** | | (0.270) | -0.196 | |  | | | | (0.214) | |
| Latent variable |  |  |  |  |  | |  | -0.315 | | *** | | | | (0.121) | |
| Facebook profile picture perk ($\beta_{7}$) |  | |  |  | | |  |  | |  | | | | | |
| Age | -0.005 |  | (0.009) | -0.009 |  | | (0.009) | -0.006 | |  | | | (0.009) | | |
| Female | -0.034 |  | (0.216) | -0.115 |  | | (0.238) | -0.102 | |  | | | (0.209) | | |
| Cohabitating partnership | -0.013 |  | (0.253) | -0.310 |  | | (0.250) | -0.068 | |  | | | (0.243) | | |
| Number of children | -0.009 |  | (0.099) | -0.024 |  | | (0.101) | -0.034 | |  | | | (0.095) | | |
| High education | -0.667 | *** | (0.231) | -0.025 |  | | (0.242) | -0.731 | | *** | | | (0.228) | | |
| Employed | 0.055 |  | (0.242) | -0.057 |  | | (0.265) | -0.001 | |  | | | (0.237) | | |
| Buys green energy | -0.282 |  | (0.290) | -0.163 |  | | (0.334) | -0.552 | | * | | | (0.311) | | |
| Political orientation | 0.109 | ** | (0.048) | 0.087 |  | | (0.058) | 0.111 | | ** | | | (0.049) | | |
| High income | -0.071 |  | (0.251) | 0.592 | * | | (0.306) | 0.001 | |  | | | (0.242) | | |
| Latent variable |  |  |  |  |  | |  | -0.384 | | *** | | | (0.122) | | |
|  |  | |  |  | | |  |  | |  | | | | | |
| *Elements of* $\Gamma$ |  | |  |  | | |  | *Structural equation for latent variable model:* | | | | | | | |
| 85,000 households, 85,000 households | | |  | 1.007 | | *** | (0.137) | Age | 0.003 | |  | | | | (0.005) |
| 85,000 households, 130,000 households | | |  | 1.809 | | *** | (0.184) | Female | -0.149 | |  | | | | (0.142) |
| 85,000 households, 20% of coastline | | |  | -0.136 | |  | (0.147) | Cohabit. part. | -0.077 | |  | | | | (0.159) |
| 85,000 households, 30% of coastline | | |  | -0.112 | |  | (0.169) | No. of children | -0.081 | |  | | | | (0.055) |
| 85,000 households, cost | | |  | 0.108 | |  | (0.108) | High educ. | -0.150 | |  | | | | (0.138) |
| 85,000 households, letter with contribution | | |  | 0.155 | |  | (0.166) | Employed | -0.077 | |  | | | | (0.159) |
| 85,000 households, Facebook profile picture | | |  | 0.357 | | * | (0.190) | Buys green en. | -0.731 | | *** | | | | (0.156) |
| 130,000 households, 130,000 households | | |  | 0.128 | |  | (0.215) | Pol. orientation | 0.001 | |  | | | | (0.030) |
| 130,000 households, 20% of coastline | | |  | -0.283 | |  | (0.189) | High income | 0.197 | |  | | | | (0.145) |
| 130,000 households, 30% of coastline | | |  | -0.358 | |  | (0.246) |  | |  | | | | | |
| 130,000 households, cost | | |  | 0.461 | | *** | (0.099) | *Measurement equation parameters:* | | | | | | | |
| 130,000 households, letter with contribution | | |  | -0.741 | | *** | (0.187) | $\zeta_{1}$ | 3.033 | | *** | | | | (0.612) |
| 130,000 households, Facebook profile picture | | |  | -0.641 | | ** | (0.276) | $\zeta_{2}$ | 1.299 | | *** | | | | (0.289) |
| 20% of coastline, 20% of coastline | | |  | 0.691 | | *** | (0.140) | $\zeta_{3}$ | 0.998 | | *** | | | | (0.239) |
| 20% of coastline, 30% of coastline | | |  | 0.904 | | *** | (0.201) | $\zeta_{4}$ | 1.807 | | *** | | | | (0.288) |
| 20% of coastline, cost | | |  | -0.049 | |  | (0.097) | $\zeta_{5}$ | 1.621 | | *** | | | | (0.292) |
| 20% of coastline, letter with contribution | | |  | -0.073 | |  | (0.223) | $\zeta_{6}$ | 1.284 | | *** | | | | (0.241) |
| 20% of coastline, Facebook profile picture | | |  | 0.057 | |  | (0.232) | $\zeta_{7}$ | 0.398 | | *** | | | | (0.152) |
| 30% of coastline, 30% of coastline | | |  | -0.333 | |  | (0.214) |  | | |  | | | | |
| 30% of coastline, cost | | |  | -0.445 | | *** | (0.105) |  | | | | | | | |
| 30% of coastline, letter with contribution | | |  | -0.035 | |  | (0.314) | $\tau_{1}$ | -3.533 | | *** | | | | (1.090) |
| 30% of coastline, Facebook profile picture | | |  | 0.433 | |  | (0.329) | $\delta_{11}$ | 4.921 | | *** | | | | (0.725) |
| cost, cost | | |  | 0.812 | | *** | (0.074) | $\delta_{21}$ | 3.968 | | *** | | | | (0.675) |
| cost, letter with contribution | | |  | 0.446 | | ** | (0.192) | $\tau_{2}$ | -1.215 | | *** | | | | (0.442) |
| cost, Facebook profile picture | | |  | 0.623 | | *** | (0.209) | $\delta_{12}$ | 3.010 | | *** | | | | (0.288) |
| letter with contribution, letter with contribution | | |  | 0.311 | |  | (0.375) | $\delta_{22}$ | 2.414 | | *** | | | | (0.362) |
| letter with contribution, Facebook profile picture | | |  | 0.527 | |  | (0.363) | $\tau_{3}$ | -1.721 | | *** | | | | (0.363) |
| Facebook profile picture, Facebook profile picture | | |  | -0.296 | |  | (0.401) | $\delta_{13}$ | 2.705 | | *** | | | | (0.236) |
|  | | |  |  | |  |  | $\delta_{23}$ | 2.231 | | *** | | | | (0.284) |
|  | | |  |  | |  |  | $\tau_{4}$ | -0.936 | |  | | | | (0.579) |
|  | | |  |  | |  |  | $\delta_{14}$ | 4.273 | | *** | | | | (0.425) |
|  | | |  |  | |  |  | $\delta_{24}$ | 2.438 | | *** | | | | (0.614) |
|  | | |  |  | |  |  | $\tau_{5}$ | -2.614 | | *** | | | | (0.514) |
|  | | |  |  | |  |  | $\delta_{15}$ | 3.964 | | *** | | | | (0.401) |
|  | | |  |  | |  |  | $\delta_{25}$ | 3.156 | | *** | | | | (0.445) |
|  | | |  |  | |  |  | $\tau_{6}$ | -0.626 | |  | | | | (0.414) |
|  | | |  |  | |  |  | $\delta_{16}$ | 3.705 | | *** | | | | (0.359) |
|  | | |  |  | |  |  | $\delta_{26}$ | 1.328 | | *** | | | | (0.380) |
|  | | |  |  | |  |  | $\tau_{7}$ | 0.238 | |  | | | | (0.168) |
|  | | |  |  | |  |  | $\delta_{17}$ | 2.630 | | *** | | | | (0.261) |
|  | | |  |  | |  |  | $\delta_{27}$ | 1.720 | | *** | | | | (0.523) |
|  | | |  |  | |  |  |  |  | |  | | | |  |

Note: ***, **, * denote significance at the 1%, 5%, and 10% level, respectively.
