# Supplementary material for: Modelling welfare estimates in discrete choice experiments for seaweed-based renewable energy
Source: PLoS One. 2021 Nov 29;16(11):e0260352. doi: 10.1371/journal.pone.0260352 (PMC8629263; doi:10.1371/journal.pone.0260352)
Supplement: S2 File — (DOCX) [file pone.0260352.s002.docx]

SUPPORTING INFORMATION

S2 QUESTIONNAIRE

We’d like to invite you to take part in a survey conducted by Queen’s University Belfast on the use of seaweed as a form of renewable energy. The results of this survey will be used for statistical analyses and to inform researchers and policymakers on people’s preferences for green energy. All responses are anonymous and confidential and will not be used for any other purpose.

Your household is among a number of households across the UK being randomly selected to participate in this research, so your participation is very important.

Please note that there are no right or wrong answers; we are only interested in your opinion. Often, when talking about green energy, or climate change, people feel pressured to say they care more about the environment than they actually do. For this survey to be effective, it’s important that you respond freely and sincerely. This questionnaire should take about 20 minutes.

If you have any questions about this research, you can contact Dr. XXXX at XXXX.

I give informed consent to participate in this study.

🗆 Yes

🗆 No, I do not want to participate.

**Screening Questions**

1) What country do you live in?

| England | 🗆 |
| --- | --- |
| Northern Ireland | 🗆 |
| Scotland | 🗆 |

2) What year were you born? (YYYY)

_________________________

3) What gender are you?

| Male | 🗆 |
| --- | --- |
| Female | 🗆 |

First, we would like to ask you some general questions about the environment and climate change.

1) How important is protecting the environment to you personally?

| Very important | 🗆 |
| --- | --- |
| Fairly important | 🗆 |
| Not very important | 🗆 |
| Not at all important | 🗆 |
| Don’t know | 🗆 |

2) In general, do you consider that you are very well, fairly well, fairly badly or very badly informed about environmental issues?

| Very well informed | 🗆 |
| --- | --- |
| Fairly well informed | 🗆 |
| Fairly badly informed | 🗆 |
| Very badly informed | 🗆 |
| Don’t know | 🗆 |

3) In your opinion, to what extent do the following factors influence your quality of life?

|  |  | Very much | Quite a lot | Not much | Not at all | Don’t know |
| --- | --- | --- | --- | --- | --- | --- |
| 1 | State of the environment | 🗆 | 🗆 | 🗆 | 🗆 | 🗆 |
| 2 | Economic factors | 🗆 | 🗆 | 🗆 | 🗆 | 🗆 |
| 3 | Social factors | 🗆 | 🗆 | 🗆 | 🗆 | 🗆 |

4) Please tell us to what extent you agree or disagree with each of the following statements.

|  |  | Totally agree | Tend to agree | Tend to disagree | Totally disagree | Don't know |
| --- | --- | --- | --- | --- | --- | --- |
| 1 | You are willing to buy environmentally friendly products even if they cost a little bit more. | 🗆 | 🗆 | 🗆 | 🗆 | 🗆 |
| 2 | As an individual you can play a role in protecting the environment in your country. | 🗆 | 🗆 | 🗆 | 🗆 | 🗆 |
| 3 | The big polluters should be mainly responsible for making good the environmental damage they cause. | 🗆 | 🗆 | 🗆 | 🗆 | 🗆 |
| 4 | Environmental issues have a direct effect on your daily life. | 🗆 | 🗆 | 🗆 | 🗆 | 🗆 |

5) In your opinion, is each of the following currently doing too much, doing about the right amount, or not doing enough to protect the environment?

|  | | Doing too much | Doing about the right amount | Not doing enough | Don't know |
| --- | --- | --- | --- | --- | --- |
| 1 | Big companies and industry | 🗆 | 🗆 | 🗆 | 🗆 |
| 2 | Citizens themselves | 🗆 | 🗆 | 🗆 | 🗆 |
| 3 | Your city, town or village | 🗆 | 🗆 | 🗆 | 🗆 |
| 4 | Your region | 🗆 | 🗆 | 🗆 | 🗆 |
| 5 | The government | 🗆 | 🗆 | 🗆 | 🗆 |

6) Which of the following do you consider to be the single most serious problem facing the world as a whole?

| Climate change | 🗆 |
| --- | --- |
| International terrorism | 🗆 |
| Poverty, hunger and lack of drinking water | 🗆 |
| Spread of infectious diseases | 🗆 |
| The economic situation | 🗆 |
| Proliferation of nuclear weapons | 🗆 |
| Armed conflicts | 🗆 |
| The increasing global population | 🗆 |
| Other | 🗆 |
| None | 🗆 |
| Don't know | 🗆 |

7) And how serious a problem do you think climate change is at this moment? Please use a scale from 1 to 10, with ‘1’ meaning it is “not at all a serious problem” and ‘10’ meaning it is “an extremely serious problem”.

| 1 Not at all a serious problem | | | | | 10 An extremely serious problem | | | | | Don't know |
| --- | --- | --- | --- | --- | --- | --- | --- | --- | --- | --- |
| 1 | 2 | 3 | 4 | 5 | 6 | 7 | 8 | 9 | 10 | 11 |

8) In your opinion, who is responsible for tackling climate change? (Tick all that apply.)

| National governments | 🗆 |
| --- | --- |
| Regional and local authorities | 🗆 |
| Business and industry | 🗆 |
| You personally | 🗆 |
| Environmental groups | 🗆 |
| Other | 🗆 |
| None | 🗆 |
| Don't know | 🗆 |

9) How important do you think it is that the government sets targets to increase the amount of renewable energy used, such as wind or solar power, by 2030?

| Very important | 🗆 |
| --- | --- |
| Fairly important | 🗆 |
| Not very important | 🗆 |
| Not at all important | 🗆 |
| Don’t know | 🗆 |

Recent data show that the UK has decreased greenhouse gas emissions by 38% since 1990. The 2030 target is to decrease them a total of 57%. One way of reaching this target is by using more renewable energy.

10) Overall, how much do you favour the use of renewable energy as one of the ways to provide energy for the UK or for Northern Ireland?

| **For the UK** | |  | **For Northern Ireland** | |
| --- | --- | --- | --- | --- |
| Strongly favour | 🗆 |  | Strongly favour | 🗆 |
| Somewhat favour | 🗆 |  | Somewhat favour | 🗆 |
| Somewhat oppose | 🗆 |  | Somewhat oppose | 🗆 |
| Strongly oppose | 🗆 |  | Strongly oppose | 🗆 |
| Don’t know | 🗆 |  | Don’t know | 🗆 |

Some people are worried about supporting renewable energy because it requires a lot of land. For example, for the same amount of energy you would get from 1 nuclear power plant, you would need a solar plant at least 35 times the size of the nuclear plant, or a wind farm at least 200 times the size of the nuclear plant.

11) How important is protecting land to you?

| Very important | 🗆 |
| --- | --- |
| Fairly important | 🗆 |
| Not very important | 🗆 |
| Not at all important | 🗆 |
| Don’t know | 🗆 |

**A New Form of Renewable Energy**

Recently, scientists have studied using seaweed as a source of renewable energy. Seaweed farms can be grown in salt water, from long lines supported by buoys and are harvested once a year. The seaweed is treated through a biological process, which generates methane gas. The methane can be passed through an engine to produce renewable energy.

The potential benefit of seaweed farming is that there is no competition with land resources to grow the crop, therefore no chemical fertilizers and there is improved waste processing. However, seaweed farms may hinder recreational use at a specific site, might affect the local fishermen and the view of the buoys might bother some people.

1) Overall, how much would you favour the use of seaweed to produce renewable energy?

| Strongly favour | 🗆 |
| --- | --- |
| Somewhat favour | 🗆 |
| Somewhat oppose | 🗆 |
| Strongly oppose | 🗆 |
| Don’t know | 🗆 |

**Analysing the different characteristics of renewable energy**

The UK government is considering a program which uses seaweed for renewable energy. This program can be described using 4 characteristics. I will describe each characteristic here.

1) **Number of households powered using seaweed.**

The renewable energy created from the seaweed farms would be used to power households. Seaweed could be farmed on a smaller scale, powering 45,000 households a year, on a medium scale, or on a larger scale, powering 130,000 households a year.

| **Number of households powered** | 45,000 Households | 85,000 Households | 130,000 Households |
| --- | --- | --- | --- |

How much would you favour your own household being powered by renewable energy from seaweed?

| Strongly favour | 🗆 |
| --- | --- |
| Somewhat favour | 🗆 |
| Somewhat oppose | 🗆 |
| Strongly oppose | 🗆 |
| Don’t know | 🗆 |

2) **Percentage of coastline used for seaweed farms**

The seaweed would be grown along the UK coastline. In some areas, seaweed farms could be extended further into the sea, so the percentage of coastline used could vary. The percentage could be as low as 10% or as high as 30%.

How concerned would you be with using sea water sites for growing seaweed?

| Very concerned | 🗆 |
| --- | --- |
| Fairly concerned | 🗆 |
| Not very concerned | 🗆 |
| Not at all concerned | 🗆 |
| Don’t know | 🗆 |

3) **Cost**

The use of seaweed to generate green bioenergy, however, may come with an additional cost to cover seaweed farming costs (until improved technology and mechanization brings the costs down). Households would, therefore, have to opt into a seaweed project scheme on their electricity bill in order to support the additional costs. The **cost** would be an additional cost on top of your current household annual electricity bill and could take on the following values:

| **Increase in electricity bill per year** | £10 | £20 | £50 | £100 | £150 |
| --- | --- | --- | --- | --- | --- |

How much does your household currently spend per year on electricity bills? £________

4) **Perks**

Some perks might be offered if you choose to opt into the seaweed project scheme. Two perks being considered are: 1) a special eco-friendly overlay for your Facebook profile picture certified by the electricity provider, 2) a letter informing you of the number of houses powered thanks to your contribution, or 3) no perks at all.

| **Perks** | Facebook profile picture overlay | A letter with your contribution | Nothing. |
| --- | --- | --- | --- |

How much are these perks likely to affect your decision to opt into the seaweed project scheme?

|  | None | Very little | Some | A lot | Don’t know |
| --- | --- | --- | --- | --- | --- |
| Facebook profile picture overlay | 🗆 | 🗆 | 🗆 | 🗆 | 🗆 |
| A letter describing your contribution | 🗆 | 🗆 | 🗆 | 🗆 | 🗆 |

**Choice Questions**

In this part of the questionnaire, we will ask you to choose between options representing different hypothetical choices for renewable energy. Each alternative is described by the characteristics just presented to you: number of households powered, percentage of coastline used for seaweed farming, increase in electricity bill per year, and perks.

Although there might be other important factors related to the issue, remember to focus on the characteristics of the alternatives presented since they are the objects of this study. All responses are **anonymous and confidential.** **There are no right or wrong answers**, we are only interested in knowing your opinion. This survey is hypothetical and in no way binds you to any future payments. It’s only important to analyse each choice set and choose the option you prefer. Two alternatives are presented in each choice together with a “neither” option which means no change from your current situation. We will show you 10 choice questions. Treat each choice question as independent.

Before choosing your preferred option in each card, think of your household’s budget and the impact that your choice would have on your budget.

**Choice 1**

| **Characteristics** | **Alternative A** | **Alternative B** | **Neither** |
| --- | --- | --- | --- |
| **Number of households powered** | 85,000 Households | 130,000 Households | No change from your current situation |
| **% of coastline used for seaweed farms** | 10% of coastline | 20% of coastline |  |
| **Increase in electricity bill per year** | £50 | £150 |  |
| **Perks** | Facebook profile picture overlay | A letter with your contribution |  |
| **Which alternative would you choose?** | **🗆** | **🗆** | **🗆** |

**Choice 2**

| **Characteristics** | **Alternative A** | **Alternative B** | **Neither** |
| --- | --- | --- | --- |
| **Number of households powered** | 45,000 Households | 130,000 Households | No change from your current situation |
| **% of coastline used for seaweed farms** | 10% of coastline | 30% of coastline |  |
| **Increase in electricity bill per year** | £150 | £20 |  |
| **Perks** | A letter with your contribution | None |  |
| **Which alternative would you choose?** | **🗆** | **🗆** | **🗆** |

**Choice 3**

| **Characteristics** | **Alternative A** | **Alternative B** | **Neither** |
| --- | --- | --- | --- |
| **Number of households powered** | 45,000 Households | 85,000 Households | No change from your current situation |
| **% of coastline used for seaweed farms** | 30% of coastline | 20% of coastline |  |
| **Increase in electricity bill per year** | £50 | £100 |  |
| **Perks** | None | None |  |
| **Which alternative would you choose?** | **🗆** | **🗆** | **🗆** |

**Choice 4**

| **Characteristics** | **Alternative A** | **Alternative B** | **Neither** |
| --- | --- | --- | --- |
| **Number of households powered** | 85,000 Households | 45,000 Households | No change from your current situation |
| **% of coastline used for seaweed farms** | 20% of coastline | 10% of coastline |  |
| **Increase in electricity bill per year** | £20 | £50 |  |
| **Perks** | A letter with your contribution | Facebook profile picture overlay |  |
| **Which alternative would you choose?** | **🗆** | **🗆** | **🗆** |

**Choice 5**

| **Characteristics** | **Alternative A** | **Alternative B** | **Neither** |
| --- | --- | --- | --- |
| **Number of households powered** | 130,000 Households | 130,000 Households | No change from your current situation |
| **% of coastline used for seaweed farms** | 30% of coastline | 10% of coastline |  |
| **Increase in electricity bill per year** | £20 | £100 |  |
| **Perks** | Facebook profile picture overlay | None |  |
| **Which alternative would you choose?** | **🗆** | **🗆** | **🗆** |

**Choice 6**

| **Characteristics** | **Alternative A** | **Alternative B** | **Neither** |
| --- | --- | --- | --- |
| **Number of households powered** | 45,000 Households | 130,000 Households | No change from your current situation |
| **% of coastline used for seaweed farms** | 20% of coastline | 20% of coastline |  |
| **Increase in electricity bill per year** | £10 | £100 |  |
| **Perks** | A letter with your contribution | Facebook profile picture overlay |  |
| **Which alternative would you choose?** | **🗆** | **🗆** | **🗆** |

**Choice 7**

| **Characteristics** | **Alternative A** | **Alternative B** | **Neither** |
| --- | --- | --- | --- |
| **Number of households powered** | 130,000 Households | 85,000 Households | No change from your current situation |
| **% of coastline used for seaweed farms** | 20% of coastline | 30% of coastline |  |
| **Increase in electricity bill per year** | £50 | £10 |  |
| **Perks** | A letter with your contribution | A letter with your contribution |  |
| **Which alternative would you choose?** | **🗆** | **🗆** | **🗆** |

**Choice 8**

| **Characteristics** | **Alternative A** | **Alternative B** | **Neither** |
| --- | --- | --- | --- |
| **Number of households powered** | 130,000 Households | 45,000 Households | No change from your current situation |
| **% of coastline used for seaweed farms** | 30% of coastline | 10% of coastline |  |
| **Increase in electricity bill per year** | £20 | £10 |  |
| **Perks** | None | A letter with your contribution |  |
| **Which alternative would you choose?** | **🗆** | **🗆** | **🗆** |

**Choice 9**

| **Characteristics** | **Alternative A** | **Alternative B** | **Neither** |
| --- | --- | --- | --- |
| **Number of households powered** | 45,000 Households | 45,000 Households | No change from your current situation |
| **% of coastline used for seaweed farms** | 20% of coastline | 30% of coastline |  |
| **Increase in electricity bill per year** | £150 | £50 |  |
| **Perks** | Facebook profile picture overlay | Facebook profile picture overlay |  |
| **Which alternative would you choose?** | **🗆** | **🗆** | **🗆** |

**Choice 10**

| **Characteristics** | **Alternative A** | **Alternative B** | **Neither** |
| --- | --- | --- | --- |
| **Number of households powered** | 85,000 Households | 130,000 Households | No change from your current situation |
| **% of coastline used for seaweed farms** | 20% of coastline | 30% of coastline |  |
| **Increase in electricity bill per year** | £50 | £50 |  |
| **Perks** | None | Facebook profile picture overlay |  |
| **Which alternative would you choose?** | **🗆** | **🗆** | **🗆** |

**About the choice questions**

1) When you made your choices did you give more importance to one of the characteristics, or did you look at all the characteristics in the same way?

🗆 I considered all the characteristics

🗆 I considered mainly one characteristics. Which one?

| Number of households powered | 🗆 |
| --- | --- |
| Percent of coastline used for seaweed farming | 🗆 |
| Increase in electricity bill per year | 🗆 |
| Perks | 🗆 |

2) How would you rank the characteristics according to their importance? (1^st^ being the most important to you and the 4^th^ as the least important):

| Number of households powered |  |
| --- | --- |
| Percent of coastline used for seaweed farming |  |
| Increase in electricity bill per year |  |
| Perks |  |

3) Did you answer the choice questions according to what was best for society or according to what was best for you?

🗆 Society

🗆 Me

🗆 Don’t know

**Hypothetical Scenario**

Next, we would like to ask you about a hypothetical scenario.

Imagine the following situation:

1) You are in a room with 4 other people and a researcher. Each of you are given **£1** in loose change.

2) A box is passed around to each of the 5 people in the room (the researcher is excluded).

3) Each person may put all or any of the £1 into the box.

4) *(Treatment 1 (standard PGG): After the box goes around the room, the researcher* ***doubles*** *the money in the box and distributes it equally among each person in the room regardless of how much money they put into the box.)*

*(Treatment 2 (climate change): After the box goes around the room, the researcher* ***doubles*** *the money in the box which is then used to reduce CO_2_ emissions.*

*(Treatment 3 (climate change): After the box goes around the room, the researcher* ***doubles*** *the money in the box which is then used to reduce CO_2_ emissions.*

*(Treatment 4 (climate change): After the box goes around the room, the researcher* ***doubles*** *the money in the box which is then used to reduce CO_2_ emissions.*

5) *(Each person will go home with whatever they kept plus what they received from the box.)*

*(Each person will go home with whatever they kept.)*

*(Each person will go home with whatever they kept.)*

*(Each person will go home with whatever they kept.)*

*(T1: No info.)*

*(T2: No info.)*

*(T3 (Good ranking): The UK is among the top 3 countries with the* ***best*** *climate change performance index, out of 58 countries. This index measures how well a country does with respect to emissions levels and trends.)*

*(T4 (Bad ranking): The UK is among the* ***worst*** *3 countries in terms of total greenhouse gas emissions, in Europe. This is measured in million tonnes of CO_2_ equivalents per year.)*

How much of the **£1**, if any, would you put into the box? Please try to answer the question as if the money were real.

£_____

Please indicate in the *(second)* table below, how much money, if any, you would put into the box, given the average contribution of the other group members. Remember that each entry is a separate case, and for each entry, you can contribute any amount between £0 and £1.

*(The first table below is to help determine your payoff based on how much money you put into the box and the average contribution of the other group members. [Table below only shown for Treatment 1.])*


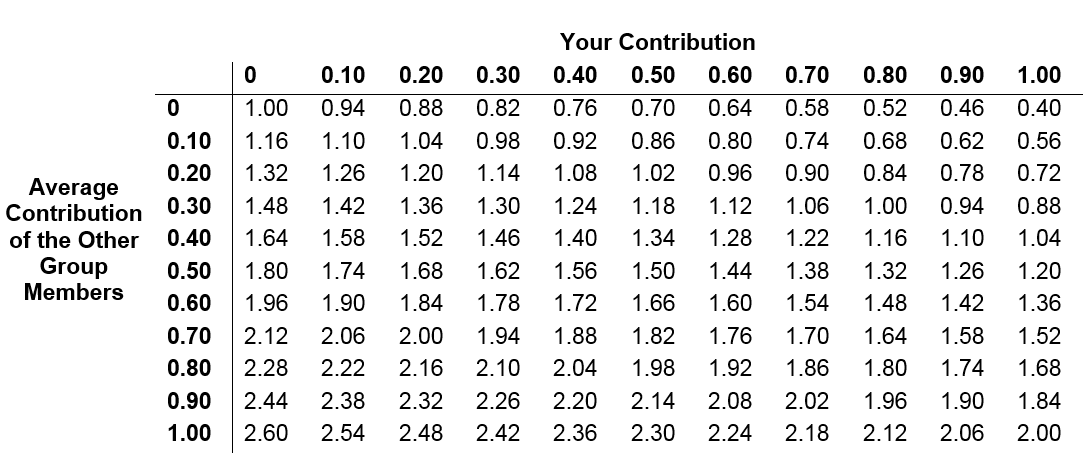


| **Average contribution of the other group members** | **Money YOU put into the box** |
| --- | --- |
| £0 |  |
| £0.10 |  |
| £0.20 |  |
| £0.30 |  |
| £0.40 |  |
| £0.50 |  |
| £0.60 |  |
| £0.70 |  |
| £0.80 |  |
| £0.90 |  |
| £1 |  |

Now imagine that you are in a room with 4 other people and a researcher, but this time you are given **£1,000**:

1) Exactly like before, a box is passed around to each of the 5 people in the room (the researcher is excluded).

2) Each person may put all or any of the £1,000 into the box.

3) *(Treatment 1 (standard PGG): After the box goes around the room, the researcher* ***doubles*** *the money in the box and distributes it equally among each person in the room regardless of how much money they put into the box.)*

*(Treatment 2 (climate change): After the box goes around the room, the researcher* ***doubles*** *the money in the box which is then used to reduce CO_2_ emissions.*

*(Treatment 3 (climate change): After the box goes around the room, the researcher* ***doubles*** *the money in the box which is then used to reduce CO_2_ emissions.*

*(Treatment 4 (climate change): After the box goes around the room, the researcher* ***doubles*** *the money in the box which is then used to reduce CO_2_ emissions.*

4) *(Each person will go home with whatever they kept plus what they received from the box.)*

*(Each person will go home with whatever they kept.)*

*(Each person will go home with whatever they kept.)*

*(Each person will go home with whatever they kept.)*

How much of the **£1,000**, if any, would you put into the box? Please try to answer the question as if the money were real.

£_____

Please indicate in the *(second)* table below, how much money, if any, you would put into the box, given the average contribution of the other group members. Remember that each entry is a separate case, and for each entry, you can contribute any amount between £0 and £1,000.

*(The first table below is to help determine your payoff based on how much money you put into the box and the average contribution of the other group members. [Table below only shown for Treatment 1.])*


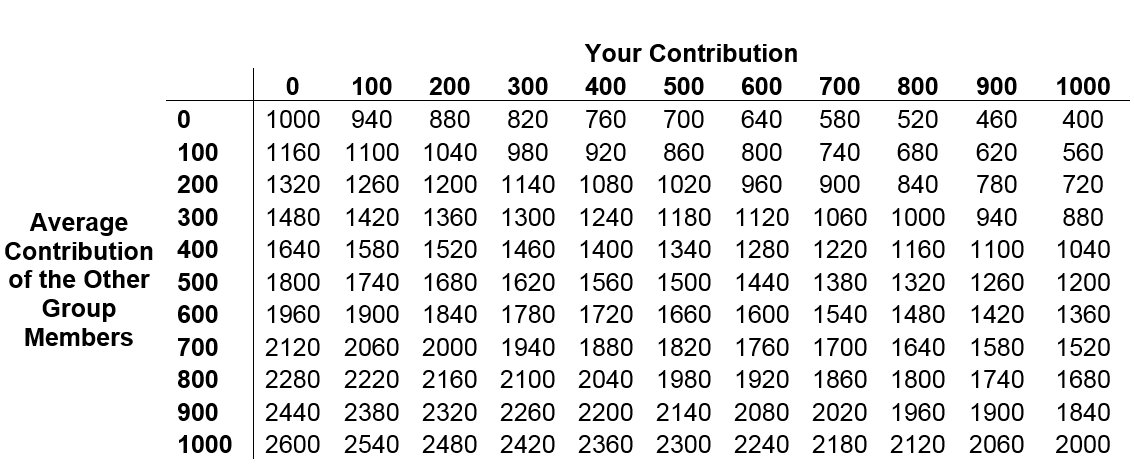


| **Average contribution of the other group members** | **Money YOU put into the box** |
| --- | --- |
| £0 |  |
| £100 |  |
| £200 |  |
| £300 |  |
| £400 |  |
| £500 |  |
| £600 |  |
| £700 |  |
| £800 |  |
| £900 |  |
| £1,000 |  |

**Some questions about yourself.**

Lastly, we would like to ask you some questions about you.

1) **Nationality:**

| English | 🗆 |
| --- | --- |
| Northern Irish | 🗆 |
| Irish | 🗆 |
| Scottish | 🗆 |
| Welsh | 🗆 |
| Other | 🗆 |

2) **Are you:**

| Single | 🗆 |
| --- | --- |
| Married | 🗆 |
| Cohabitating partnership | 🗆 |
| Divorced/Separated | 🗆 |
| Widowed | 🗆 |

3) **Structure of your household:**

Number of adults: __________

Number of children living at home: __________

4) **Highest level of education completed:**

| No education | 🗆 |
| --- | --- |
| Primary school | 🗆 |
| GCSE | 🗆 |
| A Levels | 🗆 |
| Foundation degree or equivalent | 🗆 |
| Bachelor’s degree | 🗆 |
| Master’s degree | 🗆 |
| PhD | 🗆 |

5) **Are you (a):**

| Employed full-time | 🗆 |
| --- | --- |
| Employed part-time | 🗆 |
| Self-employed | 🗆 |
| Retired | 🗆 |
| Unemployed | 🗆 |
| Homemaker/Looking after family | 🗆 |
| Student | 🗆 |

6) **Which of the following do you consider yourself?**

| Catholic | 🗆 |
| --- | --- |
| Orthodox | 🗆 |
| Protestant | 🗆 |
| Other Christian | 🗆 |
| Jewish | 🗆 |
| Muslim | 🗆 |
| Sikh | 🗆 |
| Buddhist | 🗆 |
| Hindu | 🗆 |
| Atheist (you believe there is no god) | 🗆 |
| Agnostic (you are not sure if there is a god) | 🗆 |
| Other | 🗆 |
| Prefer not to say | 🗆 |

7) **Approximately how many miles do you live from the nearest coast:** ______________ miles

8) **Do you smoke?** Yes 🗆 No 🗆

9) **Do you have a Facebook account?** Yes 🗆 No 🗆

10) **Are you, or anyone else in your household, a member of an environmental organisation?**

🗆 Yes

🗆 No

🗆 Don’t know

11) **Do you, or anyone else in your household, regularly buy “green” energy?**

🗆 Yes

🗆 No

🗆 Don’t know

12) **Do you or anyone in your household work in the energy sector?** Yes 🗆 No 🗆

13) When people talk about politics, the terms left and right are usually used. Below there is a left-right axis. **Where would you place yourself on this axis? Indicate it with an X.**

| **Left Right** | | | | | | | | | |
| --- | --- | --- | --- | --- | --- | --- | --- | --- | --- |
| **1** | **2** | **3** | **4** | **5** | **6** | **7** | **8** | **9** | **10** |

14) This survey is **anonymous** and the data will be confidential. With this in mind, could you tell me, **what is your gross (before tax) annual HOUSEHOLD income?**

| < £ 15,000 | 🗆 |
| --- | --- |
| £ 15,000- £ 23,500 | 🗆 |
| £ 23,501- £ 33,800 | 🗆 |
| £ 33,801- £ 48,000 | 🗆 |
| £ 48,001- £87,500 | 🗆 |
| £87,501+ | 🗆 |

Thank you for taking part in the study, please click "Submit" to finish the survey and record your answers.

The choices you selected will be confidential and will not be traceable to any individual by name. The data collected will only be used by the researchers who are analysing the data. By participating in the study you are helping researchers understand the value of **using seaweed as a source of green energy.**

Your data will be kept confidential and anonymous. If you have any further questions or queries please do not hesitate to contact the principal investigator Dr. XXXX (email).
